# Design, Synthesis, and Anti-Inflammatory Activities of 12-Dehydropyxinol Derivatives

**DOI:** 10.3390/molecules28031307

**Published:** 2023-01-30

**Authors:** Yunxiao Wang, Xiaoliang Mi, Yuan Du, Shuang Li, Liping Yu, Meng Gao, Xiaoyue Yang, Zhihua Song, Hui Yu, Gangqiang Yang

**Affiliations:** 1 School of Pharmacy, Collaborative Innovation Center of Advanced Drug Delivery System and Biotech Drugs in Universities of Shandong, Key Laboratory of Molecular Pharmacology and Drug Evaluation (Yantai University), Ministry of Education, Yantai University, Yantai 264005, China; 2 College of Food Engineering, Ludong University, Yantai 264025, China

**Keywords:** pyxinol, anti-inflammatory activity, ginsenosides, structure–activity relationship

## Abstract

Pyxinol skeleton is a promising framework of anti-inflammatory agents formed in the human liver from 20*S*-protopanaxadiol, the main active aglycone of ginsenosides. In the present study, a new series of amino acid-containing derivatives were produced from 12-dehydropyxinol, a pyxinol oxidation metabolite, and its anti-inflammatory activity was assessed using an NO inhibition assay. Interestingly, the dehydrogenation at C-12 of pyxinol derivatives improved their potency greatly. Furthermore, half of the derivatives exhibited better NO inhibitory activity than hydrocortisone sodium succinate, a glucocorticoid drug. The structure–activity relationship analysis indicated that the kinds of amino acid residues and their hydrophilicity influenced the activity to a great extent, as did *R*/*S* stereochemistry at C-24. Of the various derivatives, **5c** with an *N*-Boc-protected phenylalanine residue showed the highest NO inhibitory activity and relatively low cytotoxicity. Moreover, derivative **5c** could dose-dependently suppress iNOS, IL-1β, and TNF-α via the MAPK and NF-κB pathways, but not the GR pathway. Overall, pyxinol derivatives hold potential for application as anti-inflammatory agents.

## 1. Introduction

Inflammation is the physiological defense response against infection and injury. Excessive and prolonged inflammation can cause damage and result in various chronic diseases, including respiratory diseases, neurodegenerative diseases, and cancer [1,2]. Glucocorticoids (GCs) are the most effective drugs that inhibit inflammation in clinical settings; however, their side effects are extremely serious [3]. Nonetheless, owing to a lack of other effective drugs, GCs continue to be widely used to treat diseases such as COVID-19 [4]. Therefore, there is an urgent need to develop safer and more effective anti-inflammatory agents.

**Figure 1:**
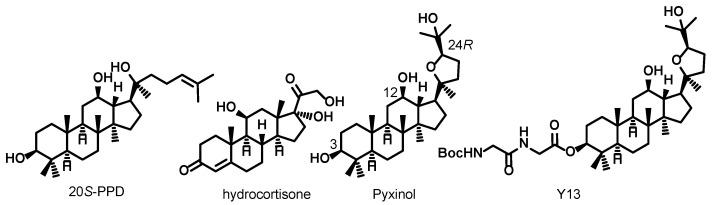
Chemical structures of 20*S*-PPD, hydrocortisone, pyxinol, and Y13.

Ginsenosides are the primary pharmacological ingredients of ginseng, the well-known orally administered herb that strengthens vitality, extends life, and supports a healthy heart [5]. To date, ginsenosides are known to possess antitumor, memory preserving, anti-inflammatory, and cardioprotective activities [5,6,7,8]. Especially, the anti-inflammatory effects are remarkable because their aglycones exhibit structural similarity with GCs [9,10]. 20*S*-Protopanoxadiol (PPD, Figure 1) is one such aglycone and has been reported to be the main intestinal metabolite of protopanoxadiol-type ginsenosides that exhibit superior anti-inflammatory activities [9,11]. Recently, pyxinol (Figure 1) has been identified as the main PPD metabolite in the human liver and has attracted special attention in drug development [12,13,14]. Pyxinol and its derivatives have been demonstrated to exert cardioprotective, antibiotic, and multidrug resistance-reversal effects [15,16,17,18,19,20,21,22,23,24,25]. Furthermore, our recent investigations have described their anti-inflammatory effects. Structure–activity relationship analysis revealed that the effects of pyxinol derivatives are closely related to *R*/*S* stereochemistry at C-24, and that the C-3 is a modifiable position for drug development [26,27]. Subsequently, derivative Y13 was identified to display better anti-inflammatory activities than hydrocortisone sodium succinate (HSS), the clinically approved steroid drug belonging to GCs [26]. Y13 could suppress proinflammatory cytokines to reduce inflammation via nuclear factor κB (NF-κB) and MAPK pathways, which is consistent with 20*S*-PPD and parental ginsenosides [11,28].

Previous studies have reported that the 24*R*-epimer of pyxinol is selectively metabolized by CYP3A4 in the human liver to produce oxidation metabolites [14], such as 3-dehydropyxinol or 12-dehydropyxinol [12]. As the modification at C-3 could substantially improve the anti-inflammatory activities of pyxinol derivatives and the structural diversity of amino acids, 24 new 3-amino acid-12-dehydropyxinol derivatives were prepared in this study, and their anti-inflammatory effects in lipopolysaccharide (LPS)-induced macrophages were assessed. Of these, an *N*-Boc-protected phenylalanine derivative of 12-dehydropyxinol (**5c**) was identified to be the most potent compound and was used for mechanistic studies.

## 2. Results and Discussion

### 2.1. Chemistry

**Scheme 1:**
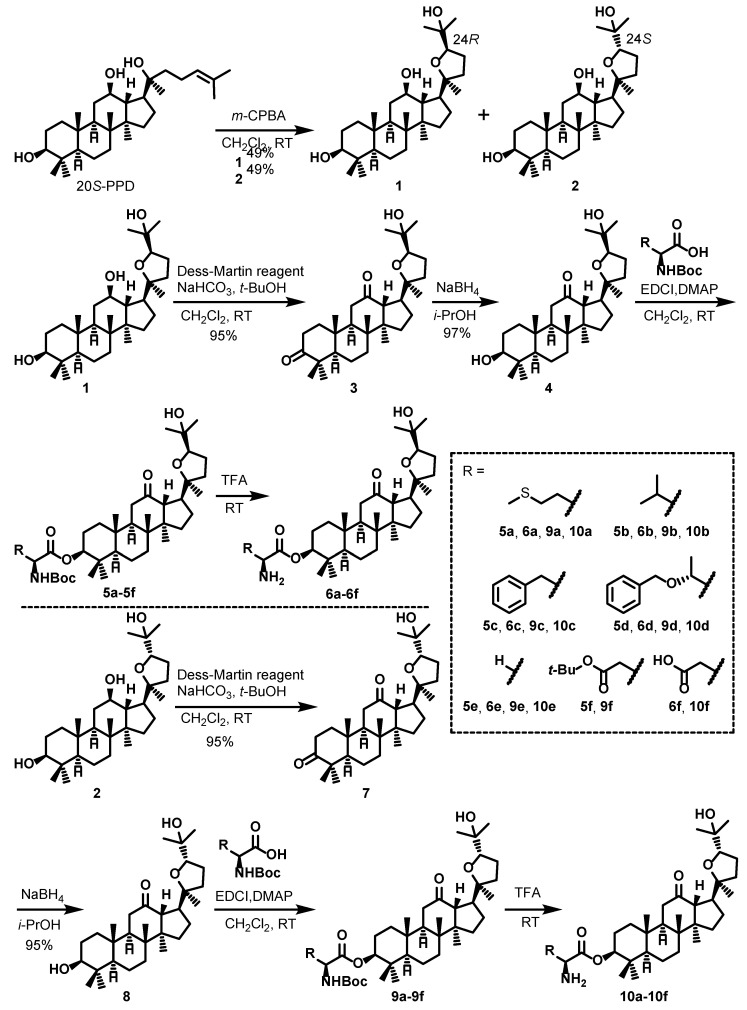
Synthesis of 12-dehydropyxinol derivatives.

As described previously [16,29], pyxinol (**1**) and 24*S*-pyxinol (**2**) were obtained via the one step epoxidation of commercial 20*S*-PPD, which is shown in Scheme 1. 3,12-Didehydropyxinol was then synthesized via Dess–Martin oxidation and was subsequently selectively reduced in NaBH_4_/isopropanol solution to obtain 12-dehydropyxinol with a yield of >90%. Amino acid-modified 12-dehydropyxinol derivatives (**5a**–**5f**) were next produced via DMAP-mediated esterification. Later, trifluoroacetic acid was utilized for *N*-Boc deprotection to obtain derivatives **6a**–**6f**. The 24*S*-epimers (**9a**–**9f**, **10a**–**10f**) were similarly synthesized from 24*S*-pyxinol.

### 2.2. NO-Inhibition of 12-Dehydropyxinol Derivatives

**Figure 2:**
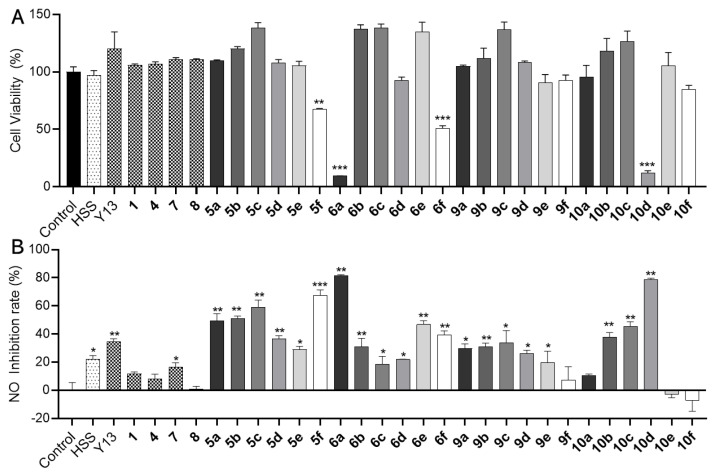
Inhibitory effects of 12-dehydropyxinol derivatives on LPS-triggered NO production in RAW264.7 macrophages: (**A**) Effect of 12-dehydropyxinol derivatives on cell survival and (**B**) LPS-triggered NO production. After pretreatment with test compounds (20 μM) for 2 h, the macrophages were treated with LPS (1 μg/mL) and further incubated for 24 h. Cell viability and nitrite concentrations were determined using MTT assays and Griess reaction, respectively. Data are expressed as the means ± SD (*n* = 3): * *p* < 0.05, ** *p* < 0.01, *** *p* < 0.001 vs. control. HSS: hydrocortisone sodium succinate.

**Figure 3:**
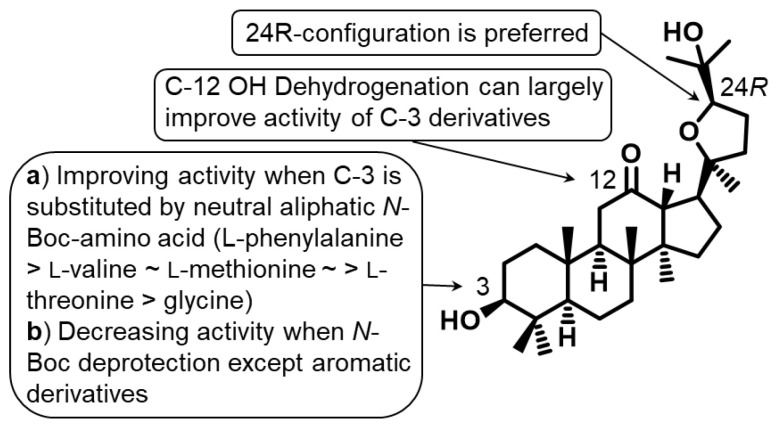
Preliminary structure–activity relationships of 12-dehydropyxinol derivatives.

The inhibitory effect of specific compounds on LPS-triggered NO production is closely related to their anti-inflammatory activity [30,31]. Herein, the NO inhibition effects of all synthesized 12-dehydropyxinol derivatives were assessed in RAW264.7 macrophages using a Griess assay with HSS as the positive control. The cytotoxicity assay showed that most of the 12-dehydropyxinol derivatives did not display any evident toxic effects on RAW264.7 cells, except **5f**, **6a**, **6f**, and **10d** when used at a concentration of 20 μM (Figure 2A). Then, these derivatives with no evident cytotoxicity were used for the NO inhibition assay. The treatment of RAW264.7 cells with 12-dehydropyxinol derivatives significantly suppressed the LPS-triggered NO production. Furthermore, most of the derivatives exhibited better NO inhibition activity than the parental compounds (**1**, **4**, **7**, and **8**) (Figure 2B), which agrees with previous results that modification at C-3 immensely improves anti-inflammatory activities [26,27]. Interestingly, most of the 12-dehydropyxinol derivatives also exhibited better NO inhibition activity than HSS, the positive control. Several of the 12-dehydropyxinol derivatives (**5a**, **5b**, **5c**, **5d**, **6e**, **10b**, and **10c**) exhibited even better NO inhibition activity than Y13, which has the highest potency among the known pyxinol derivatives with a hydroxy group at C-12. This observation suggests that dehydrogenation at C-12 of pyxinol can largely improve anti-inflammatory activity. Derivatives (**5a**–**5f** and **6e**) with a 24*R* configuration exhibited better potency than the corresponding 24*S* derivatives (**9a**–**9f** and **10e**), which is consistent with our previous findings that *R*/*S* stereochemistry at C-24 affects anti-inflammatory activity, with the 24*R*-configuration being preferred [26,27]. The modification types of amino acid residues at C-3 also largely affect their potency, with *N*-Boc-protected aromatic amino acids being preferred. This finding was quite different from pyxinol derivatives, in which *N*-Boc-protected neutral aliphatic amino acids were preferred, which suggests that the dehydrogenation at C-12 greatly influences the effect of the C-3 modification pattern on the improvement of anti-inflammatory activities. In most cases, the deprotection of Boc-protected amines reduced their anti-inflammatory effects, but not for the derivatives with an aromatic group. Based on the NO-inhibition activities of 12-dehydropyxinol derivatives, the structure–activity relationship (SAR) is summarized in Figure 3. Derivative **5c** with *N*-Boc-protected phenylalanine was identified to possess the highest NO inhibition activity and was selected for further studies.

### 2.3. Inhibition of LPS-Mediated Cytokines Production Using **5c**

**Figure 4:**
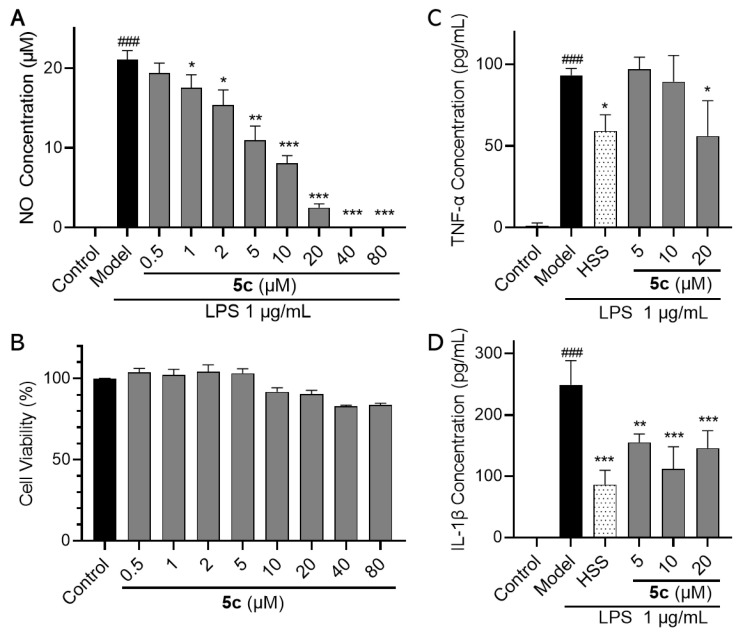
Anti-inflammatory effects of derivative **5c** on LPS-triggered RAW264.7 macrophages. The effects of derivative **5c** on (**A**) NO release, (**B**) the cytotoxicity, (**C**) TNF-α, and (**D**) IL-1β secretion. The cells were evaluated following a (**A**,**D**) 24 h or (**C**) 6-h treatment with **5c** and LPS and (**B**) after 72-h treatment with **5c**. Data are expressed as the means ± SD (*n* = 3): ^###^ *p* < 0.001 vs. control; * *p* < 0.05, ** *p* < 0.01, *** *p* < 0.001 vs. model.

To fully confirm the anti-inflammatory effects of **5c**, the concentration dependent NO-inhibition assay was first assessed. As expected, the derivative **5c** could exert a NO-inhibitory effect in a concentration-dependent manner and even exhibit a significant NO-inhibitory effect as low as 1 μM, while the derivative **5c** exhibited no evident cytotoxicity at as high as 80 μM concentration (Figure 4A,B). Interleukin-1β (IL-1β) and tumor necrosis factor-α (TNF-α) are the other known proinflammatory cytokines that are rapidly yielded in inflammation and accelerated inflammatory progression [32]. We thus next evaluated its ability to inhibit IL-1β and TNF-α production in RAW264.7 cells. LPS-triggered cells were treated with **5c** (5, 10, and 20 μM), after which the IL-1β and TNF-α levels were measured using an enzyme-linked immunosorbent assay (ELISA), which indicated a marked upregulation in the levels of IL-1β and TNF-α after LPS-induction, whereas the derivative **5c** concentration dependently suppressed this upregulation, which was even better than that of HSS (Figure 4C,D). These data further confirm the robust anti-inflammatory activities of derivative **5c**.

### 2.4. Inhibition of LPS-Mediated iNOS Upregulation and NF-κB and MAPK Activation Using **5c**

**Figure 5:**
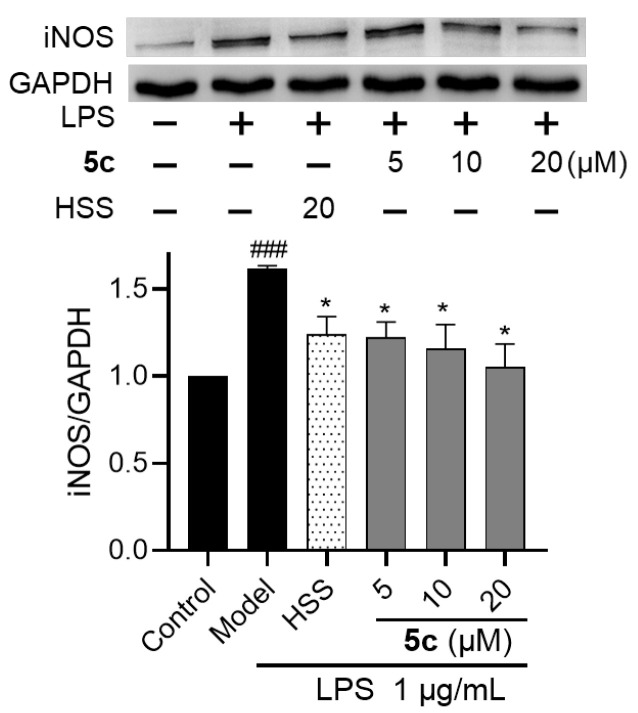
The impact of derivative **5c** on LPS-triggered iNOS protein expression in RAW264.7 macrophages following 24-h treatment with a combination of LPS (1 μg/mL) and **5c** or HSS. Data are expressed as means ± SD (*n* = 3) from triplicate experiments. ^###^ *p* < 0.001 vs. control; * *p* < 0.05 vs. model.

**Figure 6:**
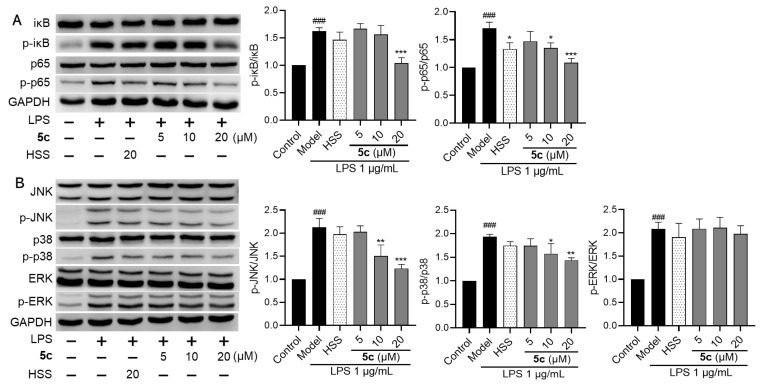
The impact of compound **5c** on LPS-triggered (**A**) NF-κB and (**B**) MAPK (JNK, p38, and ERK1/2) activation in RAW264.7 macrophages following a 2 h treatment with a combination of LPS (1 μg/mL) and **5c** or HSS. Data are expressed as the means ± SD (*n* = 3) from triplicate experiments. ^###^ *p* < 0.001 vs. control; * *p* < 0.05, ** *p* < 0.01, *** *p* < 0.001 vs. model.

Inducible nitric oxide synthase (iNOS) is the key regulator of NO synthesis in response to inflammatory stimuli and aggravates inflammatory diseases [30,33]. We thus assessed the ability of **5c** to regulate LPS-triggered iNOS expression via the 24-h treatment of RAW264.7 cells with **5c** (5, 10, and 20 μM) and LPS (1 μg/mL). The resulting iNOS level was analyzed using Western blotting. In line with the promotion of NO production, LPS induced a significant upregulation of iNOS, whereas **5c** suppressed this upregulation in a concentration-dependent manner (Figure 5). iNOS, IL-1β, and TNF-α are positively regulated in inflammation by the NF-κB pathway [34]. Both IκB and NF-κB p65 phosphorylation are key steps in the activated NF-κB pathway, which lead to the upregulation of the above inflammation-related proteins. Subsequently, the impacts of **5c** on LPS-triggered NF-κB activation were assessed. Compound **5c** inhibited LPS-triggered IκB and NF-κB p65 phosphorylation in a concentration-dependent manner, and these inhibitory effects of **5c** were better than that of HSS (Figure 6A). PPD and other ginsenosides have been confirmed to exert anti-inflammatory effects by inhibiting the NF-κB pathway, albeit weakly [9,28]. Our previous study has indicated that pyxinol might be the predominant active form of these ginsenosides that is responsible for the anti-inflammatory activity via the NF-κB pathway [27]. 24*R*-epimer of pyxinol is preferentially metabolized in the human liver via oxidation, with dehydrogenation being one of the main forms of oxidation [12,13]. Together, these results, therefore, suggest that 12-dehydropyxinol derivatives may be the active forms of ginsenosides in humans, which exert anti-inflammatory effects and prevent the upregulation of inflammatory proteins by inhibiting NF-κB activation.

The MAPK pathway is another major pathway that is activated to upregulate inflammatory proteins, such as iNOS and IL-1β, in inflammatory stimuli [35]. c-Jun N-terminal kinase (JNK), extracellular regulated protein kinases (ERK1/2), and p38 are the main signaling intermediaries in the MAPK pathway and are involved in the anti-inflammatory activity of pyxinol [26]. The impact of **5c** on the LPS-activated MAPK pathway was then assessed. LPS was observed to activate the MAPK pathway via a marked upregulation of JNK, ERK1/2, and p38 phosphorylation. On the contrary, **5c** inhibited this upregulation of JNK and p38 phosphorylation in a concentration-dependent manner. However, **5c** did not inhibit the upregulation of ERK1/2 phosphorylation (Figure 6B). Our previous study indicated that pyxinol derivatives could only inhibit the LPS-induced upregulation of ERK1/2 and p38 phosphorylation [26]. Other ginsenosides have also been reported to selectively modulate JNK phosphorylation to suppress inflammation [6,36]. The data suggest that these signaling intermediaries of MAPK may interact with these compounds and that the dehydrogenation at C-12 may greatly affect this interaction. This issue needs to be resolved in the future. In summary, compound **5c** exhibited its anti-inflammatory activity via the inhibition of JNK and p38 activation.

### 2.5. The GR-Independent Effect of **5c** on Its Anti-Inflammatory Activity

**Figure 7:**
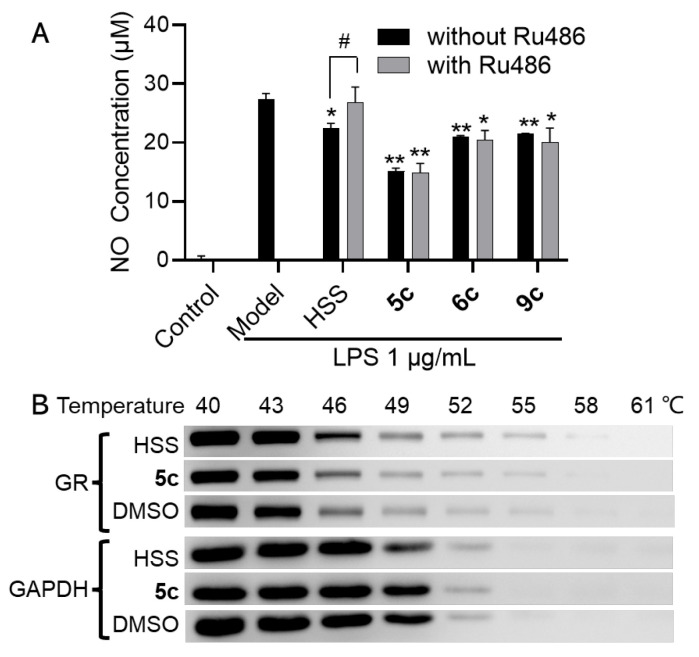
The GR-independent effect of derivative **5c** on its anti-inflammatory activity: (**A**) Effect of Ru486 (a specific GR antagonist) on the NO inhibitory effects of 12-dehydropyxinol derivatives. After pretreatment with the test compounds (20 μM) in the presence or absence of Ru486 (10 μM) for 2 h, the macrophages were treated with LPS (1 μg/mL) for another 24 h. Data are expressed as the means ± SD (*n* = 3): * *p* < 0.05, ** *p* < 0.01 vs. model; ^#^ *p* < 0.05 vs. without Ru486 group. (**B**) CESTA assessment of derivative **5c** in RAW264.7 macrophages. Immunoblotting results for GR thermal aggregation curves from samples treated with **5c** (20 μM), HSS (20 μM), or DMSO as a control.

GCs are well-known anti-inflammatory drugs in clinical settings that regulate anti-inflammation by activating the GC receptor (GR). Previous studies have reported that the activation of GR signaling is involved in the anti-inflammatory activity of ginsenosides [10,37]. Whether the anti-inflammatory activity of **5c** was related to the activation of GR signaling was assessed next. As expected, the inhibitory effect of HSS on LPS-triggered NO production was completely blocked by RU-486, a specific GR antagonist, whereas the NO inhibition effect of **5c** was not impacted by it (Figure 7A). Similar results were observed for other 12-dehydropyxinol derivatives. To further confirm whether an in situ interaction existed between GR and **5c**, the cellular thermal shift assay (CETSA) was performed. CETSA is a powerful label-free method used to analyze protein–compound interactions in physiological environments. Such interactions in situ can increase/decrease a protein’s overall resistance to thermal denaturation, thereby resulting in the amount change of the protein in the soluble lysate fraction at a high temperature [38,39]. Briefly, RAW264.7 cells were incubated with either **5c** (20 μM) or HSS (20 μM), followed by thermal denaturation at indicated temperatures. Then, the cells were lysed with freeze–thaw cycles, and soluble fractions were collected and evaluated using Western blotting. Compared with DMSO-treatment, HSS-treatment stabilized GR at high temperatures, which proves the expected interactions between GR and HSS (Figure 7B). In contrast, **5c**-treatment did not exhibit any detectable differences from DMSO-treatment at the same conditions. Molecular docking analysis also suggested that HSS had a strong binding affinity for GR; however, **5c** did not have this affinity (data not shown). These results strongly imply that **5c** exerts its anti-inflammatory activity in GR-independent ways.

## 3. Materials and Methods

### 3.1. Chemistry

The experimental reagents were obtained from commercial sources and the synthesized compounds were purified using 200–300 mesh-silica gel column chromatography. ^1^H and ^13^C NMR analyses were performed on a JEOL-ECA400 spectrometer. Chemical shifts (δ) were given as p.p.m. relative to tetramethylsilane (^1^H: 0.0 p.p.m.) in CDCl_3_ (^13^C: 77.0 p.p.m.) or CD_2_HOD in CD_3_OD (^1^H: 3.30 p.p.m., ^13^C: 49.0 p.p.m.). Thermo Scientific Q Exactive and SGW-3 apparatuses were, respectively, used to determine high-resolution mass-spectra (HRMS) and optical rotation.

#### The Synthesis of **5a**–**5f**, **6a**–**6f**, **9a**–**9f** and **10a**–**10f**

Pyxinol (**1**), 24*S*-pyxinol (**2**), and the intermediates (**3**–**4**) were prepared following published procedures [16,27,29].

To the mixture of **1** or **2** (44.0 mg, 0.0922 mmol) and NaHCO_3_ (46.4 mg, 0.553 mmol) in dehydrated CH_2_Cl_2_ (2.0 mL) was added Dess–Martin reagent (196 mg, 0.461 mmol) and *tert*-BuOH at 0 °C. Subsequent to stirring for 6 h at rt, Na_2_SO_3_ aq. was added to the mixture and extracted with EtOAC, dried over Na_2_SO_4_, and purified using column chromatography (V _petroleum ether_: V _ethyl acetate_ = 10:1–5:1–4:1) to produce **3** or **7** (over 95%).

To **3** or **7** (31 mg, 0.065 mmol) in *i*PrOH (1.2 mL), NaBH_4_ (6 mg, 0.16 mmol) was added at 0 °C under argon. Subsequent to stirring for 1 h, sat. NH_4_Cl aq. was added to the reaction mixture and extracted with EtOAc, dried over Na_2_SO_4_, and purified using column chromatography (V _petroleum ether_: V _ethyl acetate_ = 8:1–4:1–3:1) to produce **4** or **8** (over 95%).

To the mixture of **4** or **8** (20 mg, 0.042 mmol), *N*-Boc amino acid (l-*N*-Boc-Methionine, l-*N*-Boc-Valine, l-*N*-Boc-Phenylalanine, l-*N*-Boc-*O*-Bz-Threonine, *N*-Boc-Glycine, or l-*N*-Boc-*O*-*t*Bu-Aspartic acid) (0.064 mmol) and EDCI (32 mg, 0.167 mmol) in dehydrated CH_2_Cl_2_ (1.2 mL), DMAP (1 mg, 0.008 mmol) was added at 0 °C under argon. Subsequent to stirring for 1 d at rt, water was added to the reaction mixture and extracted with CH_2_Cl_2_, dried over Na_2_SO_4_, and purified using column chromatography (V _petroleum ether_: V _ethyl acetate_) to produce **5a**–**5f** and **9a**–**9f**.

TFA (1.2 mL) was added into **5a** (**5b**, **5c**, **5d**, **5e**, **5f**, **9a**, **9b**, **9c**, **9d**, **9e,** or **9f**) (0.03 mmol) at 0 °C. Subsequent to stirring at rt for 15 min, the solution was concentrated in vacuo to produce **6a**–**6f** and **10a**–**10f** (Supplementary Material).

Compound **5a**, yield: 92%. ${\left[\alpha \right]}_{D}^{21}$ + 19.4 (*c* 1.0, CHCl_3_); ^1^H NMR (400 MHz, CDCl_3_) δ 5.11 (d, J *=* 7.7 Hz, 1H, -NH-), 4.52 (dd, J = 11.0, 5.2 Hz, 1H, -COOCH-), 4.42–4.37 (m, 1H, -OCOCH-), 3.70 (dd, J = 8.4, 6.2 Hz, 1H, -OCH-), 2.90 (d, J = 9.3 Hz, 1H, -COCH-), 2.60–2.51 (m, 3H), 2.20 (d, J = 8.2 Hz, 2H), 2.14 (dd, J = 10.4, 8.0 Hz, 1H), 2.10 (s, 3H, -SCH_3_), 1.95–0.80 (m, 20H), 1.44 (s, 9H, -C(CH_3_)_3_), 1.21 (s, 3H, -CH_3_), 1.20 (s, 3H, -CH_3_), 1.11 (s, 6H, -CH_3_ × 2), 0.96 (s, 3H, -CH_3_), 0.89 (s, 3H, -CH_3_), 0.87 (s, 3H, -CH_3_), 0.76 (s, 3H, -CH_3_). ^13^C NMR (100 MHz, CDCl_3_) δ 211.2, 171.9, 155.3, 85.2, 83.6, 81.9, 79.9, 71.1, 57.0, 55.8, 55.7, 54.1, 53.2, 42.6, 40.4, 39.7, 38.1, 38.0, 37.5, 35.1, 34.1, 32.5, 32.0, 28.3 (3C), 28.1, 28.0, 27.6, 26.5, 25.6, 25.0, 24.4, 23.5, 18.2, 16.6, 16.5, 16.0, 15.6, 15.5. HRMS (ESI) *m*/*z* calcd C_40_H_67_NO_7_SNa^+^ [M + Na]^+^ 728.4530, found 728.4525.

Compound **5b**, yield: 72%. ${\left[\alpha \right]}_{D}^{22}$ + 31.1 (*c* 1.0, CHCl_3_); ^1^H NMR (400 MHz, CDCl_3_) δ 5.00 (d, J = 9.3 Hz, 1H, -NH-), 4.51 (dd, *J =* 11.4, 4.8 Hz, 1H, -COOCH-), 4.22 (dd, J = 9.1, 4.1 Hz, 1H, -OCOCH-), 3.69 (dd, J = 8.5, 6.0 Hz, 1H, -OCH-), 2.90 (d, J = 9.6 Hz, 1H, -COCH-), 2.57 (td, J = 10.2, 4.1 Hz, 1H, -COCHCHCH_2_-), 2.22–2.16 (m, 3H), 1.87–0.85 (m, 18H), 1.44 (s, 9H, -C(CH_3_)_3_), 1.21 (s, 3H, -CH_3_), 1.20 (s, 3H, -CH_3_), 1.11 (s, 3H, -CH_3_), 1.10 (s, 3H, -CH_3_), 0.99 (d, J = 6.9 Hz, 3H, -CH-CH_3_), 0.96 (s, 3H, -CH_3_), 0.89 (s, 3H, -CH_3_), 0.88 (d, J = 7.1 Hz, 3H, -CH-CH_3_), 0.87 (s, 3H, -CH_3_), 0.76 (s, 3H, -CH_3_). ^13^C NMR (100 MHz, CDCl_3_) δ 211.2, 172.0, 155.7, 85.1, 83.6, 81.6, 79.6, 71.1, 58.8, 57.0, 55.8, 55.7, 54.1, 42.6, 40.4, 39.7, 38.1, 37.9, 37.5, 35.1, 34.1, 32.0, 31.2, 28.3 (3C), 28.0, 27.6, 26.5, 25.6, 25.0, 24.4, 23.5, 19.3, 18.2, 17.2, 16.6, 16.6, 16.0, 15.6. HRMS (ESI) *m*/*z* calcd C_40_H_67_NO_7_Na^+^ [M + Na]^+^ 696.4810, found 696.4787.

Compound **5c**, yield: 83%. ${\left[\alpha \right]}_{D}^{27}$ + 37.3 (c 1.0, CHCl_3_); ^1^H NMR (400 MHz, CDCl_3_) δ 7.31–7.16 (m, 5H, Ar-H × 5), 4.90 (d, J = 8.5 Hz, 1H, -NH-), 4.56 (q, J = 7.0 Hz, 1H, -OCOCH-), 4.48 (dd, J = 11.4, 4.5 Hz, 1H, -COOCH-), 3.69 (dd, J = 8.7, 6.2 Hz, 1H, -OCH-), 3.12 (dd, J = 13.9, 5.9 Hz, 1H, PhCH_a_-), 3.01 (dd, J = 13.9, 6.7 Hz, 1H, PhCH_b_-), 2.89 (d, J = 9.3 Hz, 1H, -COCH-), 2.56 (td, J = 10.0, 4.1 Hz, 1H, -COCHCHCH_2_-), 2.27–2.12 (m, 2H), 1.89–0.80 (m, 18H), 1.39 (s, 9H, -C(CH_3_)_3_), 1.21 (s, 3H, -CH_3_), 1.19 (s, 3H, -*CH_3_*), 1.11 (s, 3H, -CH_3_), 1.10 (s, 3H, -CH_3_), 0.93 (s, 3H, -CH_3_), 0.81 (s, 3H, -CH_3_), 0.78 (s, 3H, -CH_3_), 0.76 (s, 3H, -CH_3_); ^13^C NMR (100 MHz, CDCl_3_) δ 211.2, 171.8, 155.1, 136.1, 129.3 (2C), 128.5 (2C), 126.9, 85.1, 83.6, 81.9, 79.8, 71.1, 57.0, 55.8, 55.7, 54.6, 54.1, 42.6, 40.4, 39.7, 38.5, 38.2, 37.9, 37.5, 35.1, 34.1, 32.0, 28.3 (3C), 27.9, 27.6, 26.5, 25.6, 25.0, 24.4, 23.3, 18.2, 16.6, 16.4, 16.0, 15.6. HRMS (ESI) *m*/*z* calcd C_44_H_67_NO_7_Na^+^ [M + Na]^+^ 744.4810, found 744.4779.

Compound **5d**, yield: 85%. ${\left[\alpha \right]}_{D}^{22}$ + 22.4 (*c* 1.00, CHCl_3_); ^1^H NMR (400 MHz, CDCl_3_) δ 7.33–7.24 (m, 5H, Ar-H × 5), 5.27 (d, J = 9.9 Hz, 1H, -NH-), 4.55 (d, J = 11.0 Hz, 1H, PhCH_a_-), 4.53 (dd, J = 12.1, 4.4 Hz, 1H, -COOCH-), 4.38 (d, J = 11.3 Hz, 1H, PhCH_b_-), 4.29 (dd, J = 9.8, 2.1 Hz, 1H, -OCOCH-), 4.20 (qd, J = 6.2, 2.0 Hz, 1H, PhCH_2_OCH-), 3.69 (dd, J = 8.4, 6.2 Hz, 1H, -OCH-), 2.89 (d, J = 9.6 Hz, 1H, -COCH-), 2.57 (td, J = 10.0, 4.1 Hz, 1H, -COCHCHCH_2_-), 2.21–2.15 (m, 2H), 1.99 (br, 1H), 1.86–1.02 (m, 17H), 1.45 (s, 9H, -C(CH_3_)_3_), 1.29 (d, J = 6.3 Hz, 3H, -CHCH_3_), 1.21 (s, 3H, -CH_3_), 1.19 (s, 3H, -CH_3_), 1.11 (s, 3H, -CH_3_), 1.10 (s, 3H, -CH_3_), 0.94 (s, 3H, -CH_3_), 0.87 (s, 3H, -CH_3_), 0.82 (s, 3H, -CH_3_), 0.76 (s, 3H, -CH_3_). ^13^C NMR (100 MHz, CDCl_3_) δ 211.2, 170.9, 156.0, 137.9, 128.2 (2C), 127.6, 127.4 (2C), 85.1, 83.5, 81.7, 79.7, 75.4, 71.1, 70.9, 58.6, 57.0, 55.7, 55.6, 54.0, 42.5, 40.4, 39.7, 38.2, 37.9, 37.5, 35.1, 34.1, 32.0, 28.3 (3C), 27.9, 27.6, 26.5, 25.6, 24.9, 24.4, 23.4, 18.2, 16.6, 16.5, 16.4, 16.0, 15.6. HRMS (ESI) *m*/*z* calcd C_46_H_71_NO_8_Na^+^ [M + Na]^+^ 788.5072, found 788.5042.

Compound **5e**, yield: 79%. ${\left[\alpha \right]}_{D}^{30}$ + 21.3 (*c* 1.0, CHCl_3_); ^1^H NMR (400 MHz, CDCl_3_) δ 5.01 (s, 1H, -NH-), 4.54 (dd, J = 11.2, 5.3 Hz, 1H, -COOCH-), 3.90 (d, J = 5.0 Hz, 2H, -OCOCH_2_NHBoc), 3.70 (dd, J = 8.7, 5.9 Hz, 1H, -OCH-), 2.89 (d, J = 9.6 Hz, 1H, -COCH-), 2.57 (td, J = 10.1, 4.3 Hz, 1H, -COCHCHCH_2_-), 2.27–2.09 (m, 2H), 1.90–0.80 (m, 18H), 1.45 (s, 9H, -C(CH_3_)_3_), 1.21 (s, 3H, -CH_3_), 1.20 (s, 3H, -CH_3_), 1.11 (s, 3H, -CH_3_), 1.10 (s, 3H, -CH_3_), 0.95 (s, 3H, -CH_3_), 0.87 (s, 3H, -CH_3_), 0.87 (s, 3H, -CH_3_), 0.76 (s, 3H, -CH_3_); ^13^C NMR (100 MHz, CDCl_3_) δ 211.3, 170.0, 155.6, 85.2, 83.5, 81.7, 79.9, 71.2, 57.0, 55.7, 55.7, 54.1, 42.6, 42.6, 40.4, 39.7, 38.2, 38.0, 37.5, 35.1, 34.1, 32.0, 28.3 (3C), 28.0, 27.6, 26.5, 25.6, 25.0, 24.3, 23.4, 18.2, 16.6, 16.4, 16.1, 15.6. HRMS (ESI) *m*/*z* calcd C_37_H_61_NO_7_Na^+^ [M + Na]^+^ 654.4340, found 654.4316.

Compound **5f**, yield: 80%. ${\left[\alpha \right]}_{D}^{30}$ + 34.7 (*c* 1.0, CHCl_3_); ^1^H NMR (400 MHz, CDCl_3_) δ 5.54 (d, J = 8.8 Hz, 1H, -NH-), 4.52–4.47 (m, 2H), 3.69 (dd, J = 8.7, 6.2 Hz, 1H, -OCH-), 2.94–2.88 (m, 2H), 2.73 (dd, J = 17.0, 4.4 Hz, 1H, -CH_a_COO*t*Bu), 2.56 (td, J = 10.1, 4.2 Hz, 1H, -COCHCHCH_2_-), 2.23–2.19 (m, 2H), 1.89–0.86 (m, 18H), 1.45 (s, 9H, -C(CH_3_)_3_), 1.43 (s, 9H, -C(CH_3_)_3_), 1.21 (s, 3H, -CH_3_), 1.19 (s, 3H, -CH_3_), 1.11 (s, 3H, -CH_3_), 1.10 (s, 3H, -CH_3_), 0.95 (s, 3H, -CH_3_), 0.87 (s, 3H, -CH_3_), 0.86 (s, 3H, -CH_3_), 0.76 (s, 3H, -CH_3_); ^13^C NMR (100 MHz, CDCl_3_) δ 211.2, 170.9, 170.3, 155.5, 85.1, 83.5, 81.9, 81.6, 79.8, 71.1, 57.0, 55.8, 55.7, 54.0, 50.2, 42.5, 40.4, 39.7, 38.1, 37.9, 37.7, 37.5, 35.1, 34.1, 32.0, 28.3 (3C), 28.0 (3C), 28.0, 27.6, 26.5, 25.6, 24.9, 24.4, 23.3, 18.2, 16.6, 16.5, 16.0, 15.6. HRMS (ESI) *m*/*z* calcd C_43_H_71_NO_9_Na^+^ [M + Na]^+^ 768.5021, found 768.4998.

Compound **6a**, yield: 90%. ${\left[\alpha \right]}_{D}^{21}$ + 38.9 (*c* 0.50, CH_3_OH); ^1^H NMR (400 MHz, CD_3_OD) δ 4.65 (dd, J = 10.0, 6.5 Hz, 1H, -COOCH-), 4.21 (t, J = 6.2 Hz, 1H, -OCOCH-), 3.72 (t, J = 7.1 Hz, 1H, -OCH-), 3.04 (d, J = 9.6 Hz, 1H, -COCH-), 2.67 (t, J = 7.4 Hz, 2H, -SCH_2_-), 2.51 (td, J = 10.0, 4.4 Hz, 1H, -COCHCHCH_2_-), 2.36 (t, J = 13.2 Hz, 1H, -COCH_a_CH-), 2.31–2.22 (m, 1H), 2.16–2.09 (m, 4H), 2.11 (s, 3H, -SCH_3_), 1.93–0.87 (m, 16H), 1.26 (s, 3H, -CH_3_), 1.11 (s, 3H, -CH_3_), 1.11 (s, 3H, -CH_3_), 1.09 (s, 3H, -CH_3_), 1.02 (s, 3H, -CH_3_), 0.95 (s, 3H, -CH_3_), 0.92 (s, 3H, -CH_3_), 0.77 (s, 3H, -CH_3_). ^13^C NMR (100 MHz, CD_3_OD) δ 214.3, 170.3, 86.6, 85.2, 85.0, 72.7, 58.2, 57.1, 56.9, 55.7, 53.0, 43.9, 41.8, 40.7, 39.2, 39.1, 38.7, 35.5, 35.3, 33.1, 30.9, 30.1, 28.6, 27.5, 26.2, 26.0, 25.8, 25.4, 24.4, 19.3, 17.1, 17.0, 16.5, 16.1, 14.9. HRMS (ESI) *m*/*z* calcd C_35_H_59_NO_5_SNa^+^ [M + Na]^+^ 628.4007, found 628.3995.

Compound **6b**, yield: 90%. ${\left[\alpha \right]}_{D}^{26}$ + 29.6 (*c* 1.0, CHCl_3_); δ ^1^H NMR (400 MHz, CDCl_3_) δ 4.59 (dd, J = 10.7, 3.8 Hz, 1H, -COOCH-), 3.88–3.83 (m, 1H), 3.74 (t, J = 7.1 Hz, 1H, -OCH-), 2.87 (d, J = 9.6 Hz, 1H, -COCH-), 2.57 (td, J = 9.7, 4.0 Hz, 1H, -COCHCHCH_2_-), 2.39–2.30 (m, 1H), 2.25–2.15 (m, 2H), 1.91–0.81 (m, 18H), 1.26 (s, 3H, -CH_3_), 1.20 (s, 6H, -CH_3_ × 2), 1.11 (s, 3H, -CH_3_), 1.09 (d, J = 6.6 Hz, 3H, -CHCH_3_), 1.07 (s, 3H, -CH_3_), 0.95 (s, 3H, -CH_3_), 0.88 (s, 3H, -CH_3_), 0.89 (d, J = 6.6 Hz, 3H, -CHCH_3_), 0.77 (s, 3H, -CH_3_). ^13^C NMR (100 MHz, CDCl_3_) δ 211.8, 167.7, 85.3, 83.9, 83.5, 71.3, 58.7, 57.0, 55.7, 55.6, 54.0, 42.4, 40.4, 39.6, 38.0, 37.8, 37.4, 34.4, 34.1, 32.0, 31.9, 28.1, 27.5, 26.9, 26.5, 25.6, 25.2, 23.6, 18.2, 18.0, 17.5, 16.8, 16.6, 15.9, 15.6. HRMS (ESI) *m*/*z* calcd C_35_H_59_NO_5_Na^+^ [M + Na]^+^ 596.4285, found 596.4260.

Compound **6c**, yield: 90%. ${\left[\alpha \right]}_{D}^{26}$ + 30.0 (*c* 1.0, CHCl_3_); ^1^H NMR (400 MHz, CDCl_3_) δ 7.34–7.24 (m, 5H, Ar-H × 5), 4.50–4.46 (m, 1H), 4.22 (dd, J = 11.3, 5.5 Hz, 1H, PhCH_2_CH-), 3.72 (t, J = 6.7 Hz, 1H, -OCH-), 3.30–3.25 (m, 2H), 2.86 (d, J = 9.3 Hz, 1H, -COCH-), 2.56 (td, J = 9.3, 2.4 Hz, 1H, -COCHCHCH_2_-), 2.18–2.16 (m, 2H), 1.90–0.66 (m, 18H), 1.19 (s, 3H, -CH_3_), 1.18 (s, 3H, -CH_3_), 1.11 (s, 3H, -CH_3_), 1.07 (s, 3H, -CH_3_), 0.91 (s, 3H, -CH_3_), 0.76 (s, 3H, -CH_3_), 0.73 (s, 3H, -CH_3_), 0.71 (s, 3H, -CH_3_). ^13^C NMR (100 MHz, CDCl_3_) δ 211.7, 168.8, 133.6, 129.3 (2C), 129.2 (2C), 127.9, 85.3, 84.1, 83.5, 71.4, 57.0, 55.7, 55.7, 54.3, 54.0, 42.5, 40.4, 39.6, 38.0, 37.7, 37.4, 36.5, 34.6, 34.1, 31.9, 27.9, 27.6, 26.9, 26.6, 25.6, 25.1, 23.8, 18.1, 16.6, 16.2, 16.0, 15.6. HRMS (ESI) *m*/*z* calcd C_39_H_59_NO_5_Na^+^ [M + Na]^+^ 644.4285, found 644.4258.

Compound **6d**, yield: 90%. ${\left[\alpha \right]}_{D}^{22}$ + 18.7 (*c* 1.00, CH_3_OH); ^1^H NMR (400 MHz, CD_3_OD) δ 7.36–7.26 (m, 5H, Ar-H × 5), 4.69 (d, J = 11.5 Hz, 1H, PhCH_a_-), 4.62 (dd, J = 10.6, 4.5 Hz, 1H, -COOCH-), 4.46 (d, J = 11.5 Hz, 1H, PhCH_b_-), 4.29 (qd, J = 6.3, 3.0 Hz, 1H, PhCH_2_OCH-), 4.13 (d, J = 2.7 Hz, 1H, -OCOCH-), 3.72 (dd, J = 8.9, 5.4 Hz, 1H, -OCH-), 3.03 (d, J = 9.3 Hz, 1H, -COCH-), 2.51 (td, J = 9.8, 4.3 Hz, 1H, -COCHCHCH_2_-), 2.35 (t, J = 13.2 Hz, 1H, -COCH_a_CH-), 2.12 (dd, J = 12.9, 3.6 Hz, 1H, -COCH_b_CH-), 1.92–0.90 (m, 18H), 1.42 (d, J = 6.3 Hz, 3H, -CHCH_3_), 1.25 (s, 3H, -CH_3_), 1.11 (s, 3H, -CH_3_), 1.11 (s, 3H, -CH_3_), 1.09 (s, 3H, -CH_3_), 1.01 (s, 3H, -CH_3_), 0.93 (s, 3H, -CH_3_), 0.82 (s, 3H, -CH_3_), 0.76 (s, 3H, -CH_3_). ^13^C NMR (100 MHz, CD_3_OD) δ 214.3, 168.8, 138.8, 129.4 (2C), 128.9, 128.8 (2C), 86.6, 85.2, 85.0, 74.0, 72.7, 72.0, 59.2, 58.2, 57.0, 56.9, 55.7, 43.9, 41.7, 40.7, 39.2, 39.0, 38.7, 35.5, 35.2, 33.0, 28.7, 27.5, 26.2, 26.0, 25.8, 25.5, 24.4, 19.3, 17.1, 17.0, 16.5, 16.4, 16.1. HRMS (ESI) *m*/*z* calcd C_41_H_64_NO_6_^+^ [M + H]^+^ 666.4728, found 666.4700.

Compound **6e**, yield: 90%. ${\left[\alpha \right]}_{D}^{26}$ + 14.5 (*c* 1.0, CHCl_3_); ^1^H NMR (400 MHz, CD_3_OD) δ 4.64 (dd, J = 10.9, 5.9 Hz, 1H, -COOCH-), 3.83 (d, J = 17.3 Hz, 1H, -OCOCH_a_-), 3.77 (d, J = 17.3 Hz, 1H, -OCOCH_b_-), 3.72 (t, J = 7.1 Hz, 1H, -OCH-), 3.04 (d, J = 9.6 Hz, 1H, -COCH-), 2.51 (td, J = 10.0, 4.2 Hz, 1H, -COCHCHCH_2_-), 2.36 (t, J = 13.3 Hz, 1H, -COCH_a_CH-), 2.12 (dd, J = 13.1, 3.7 Hz, 1H, -COCH_b_CH-), 1.91–0.85 (m, 18H), 1.25 (s, 3H, -CH_3_), 1.12 (s, 3H, -CH_3_), 1.11 (s, 3H, -CH_3_), 1.09 (s, 3H, -CH_3_), 1.02 (s, 3H, -CH_3_), 0.93 (s, 3H, -CH_3_), 0.92 (s, 3H, -CH_3_), 0.77 (s, 3H, -CH_3_). ^13^C NMR (100 MHz, CD_3_OD) δ 214.3, 165.0, 86.6, 85.2, 85.2, 72.7, 58.2, 57.1, 57.0, 55.8, 43.9, 41.8, 40.7, 40.6, 39.3, 39.1, 38.7, 35.6, 35.3, 33.1, 28.4, 27.5, 26.2, 26.0, 25.7, 25.4, 24.5, 19.3, 17.1, 16.8, 16.6, 16.1. HRMS (ESI) *m*/*z* calcd C_32_H_53_NO_5_Na^+^ [M + Na]^+^ 554.3816, found 554.3795.

Compound **6f**, yield: 90%. ${\left[\alpha \right]}_{D}^{30}$ + 30.1 (*c* 1.0, CHCl_3_); ^1^H NMR (400 MHz, CD_3_OD) δ 4.63 (dd, J = 11.0, 5.2 Hz, 1H, -COOCH-), 4.29 (dd, J = 12.9, 6.0 Hz, 1H, -OCOCH-), 3.72 (t, J = 7.1 Hz, 1H, -OCH-), 3.09–2.94 (m, 3H), 2.51 (td, J = 9.8, 4.2 Hz, 1H, -COCHCHCH_2_-), 2.36 (t, J = 13.3 Hz, 1H, -COCH_a_CH-), 2.12 (dd, J = 12.9, 3.6 Hz, 1H, -COCH_b_CH-), 1.92–0.95 (m, 18H), 1.25 (s, 3H, -CH_3_), 1.11 (s, 3H, -CH_3_), 1.11 (s, 3H, -CH_3_), 1.09 (s, 3H, -CH_3_), 1.01 (s, 3H, -CH_3_), 0.93 (s, 3H, -CH_3_), 0.89 (s, 3H, -CH_3_), 0.77 (s, 3H, -CH_3_). ^13^C NMR (100 MHz, CD_3_OD) δ 214.3, 172.6, 169.2, 86.6, 85.2, 85.0, 72.7, 58.2, 57.1, 57.0, 55.7, 50.8, 43.9, 41.8, 40.7, 39.2, 39.1, 38.7, 35.5, 35.3, 34.6, 33.0, 28.4, 27.5, 26.2, 26.0, 25.8, 25.4, 24.4, 19.3, 17.1, 16.9, 16.5, 16.1. HRMS (ESI) *m*/*z* calcd C_34_H_55_NO_7_Na^+^ [M + Na]^+^ 612.3871, found 612.3845.

Compound **9a**, yield: 87%. ${\left[\alpha \right]}_{D}^{20}$ + 30.0 (*c* 1.00, CHCl_3_); ^1^H NMR (400 MHz, CDCl_3_) δ 5.11 (d, J = 8.0 Hz, 1H, -NH-), 4.52 (dd, J = 11.0, 5.2 Hz, 1H, -COOCH-), 4.40 (dd, J = 12.2, 8.4 Hz, 1H, -OCOCH-), 3.71 (dd, J = 9.8, 5.6 Hz, 1H, -OCH-), 2.96 (d, J = 9.6 Hz, 1H, -COCH-), 2.59–2.51 (m, 3H), 2.22–2.12 (m, 4H), 2.10 (s, 3H, -SCH_3_), 1.95–0.85 (m, 18H), 1.45 (s, 9H, -C(CH_3_)_3_), 1.21 (s, 3H, -CH_3_), 1.19 (s, 3H, -CH_3_), 1.09 (s, 3H, -CH_3_), 1.04 (s, 3H, -CH_3_), 0.97 (s, 3H, -CH_3_), 0.90 (s, 3H, -CH_3_), 0.87 (s, 3H, -CH_3_), 0.75 (s, 3H, -CH_3_). ^13^C NMR (100 MHz, CDCl_3_) δ 210.8, 171.9, 155.3, 87.5, 85.2, 81.9, 79.9, 70.3, 57.2, 55.9, 55.8, 54.2, 53.2, 43.0, 40.4, 39.6, 38.1, 38.0, 37.5, 36.5, 34.2, 32.5, 31.9, 30.0, 28.3 (3C), 28.1, 27.7, 26.6, 26.2, 24.8, 23.9, 23.4, 18.2, 16.5 (2C), 16.1, 15.6, 15.5. HRMS (ESI) *m*/*z* calcd C_40_H_67_NO_7_SNa^+^ [M + Na]^+^ 728.4530, found 728.4501.

Compound **9b**, yield: 88%. ${\left[\alpha \right]}_{D}^{24}$ + 26.4(*c* 1.0, CHCl_3_); ^1^H NMR (400 MHz, CDCl_3_) δ 5.01 (d, J = 9.1 Hz, 1H, -NH-), 4.51 (dd, J = 11.5, 4.9 Hz, 1H, -COOCH-), 4.22 (dd, J = 9.2, 4.3 Hz, 1H, -OCOCH-), 3.71 (dd, J = 9.8, 5.6 Hz, 1H, -OCH-), 2.96 (d, J = 9.3 Hz, 1H, -COCH-), 2.54 (td, J = 9.9, 4.2 Hz, 1H, -COCHCHCH_2_-), 2.22–2.14 (m, 2H), 1.95–1.51 (m, 19H), 1.45 (s, 9H, -C(CH_3_)_3_), 1.21 (s, 3H, -CH_3_), 1.19 (s, 3H, -CH_3_), 1.09 (s, 3H, -CH_3_), 1.04 (s, 3H, -CH_3_), 0.99 (d, J = 6.9 Hz, 3H, -CHCH_3_), 0.97 (s, 3H, -CH_3_), 0.90 (s, 3H, -CH_3_), 0.88 (d, J = 7.15 Hz, 3H, -CHCH_3_), 0.88 (s, 3H, -CH_3_), 0.75 (s, 3H, -CH_3_). ^13^C NMR (100 MHz, CDCl_3_) δ 210.9, 172.0, 155.7, 87.5, 85.3, 81.6, 79.6, 70.3, 58.8, 57.2, 55.9, 55.8, 54.2, 43.0, 40.4, 39.6, 38.2, 37.9, 37.5, 36.5, 34.2, 31.9, 31.2, 28.3 (3C), 28.0, 27.7, 26.6, 26.2, 24.8, 23.9, 23.5, 19.3, 18.2, 17.1, 16.6 (2C), 16.0, 15.6. HRMS (ESI) *m*/*z* calcd C_40_H_67_NO_7_Na^+^ [M + Na]^+^ 696.4810, found 696.4782.

Compound **9c**, yield: 93%. ${\left[\alpha \right]}_{D}^{24}$ + 25.2(*c* 1.0, CHCl_3_); ^1^H NMR (400 MHz, CDCl_3_) δ 7.31–7.27 (m, 2H, Ar-H × 2), 7.23 (tt, J = 7.2, 1.6 Hz, 1H, Ar-H), 7.17 (dd, J = 8.0, 1.4 Hz, 2H, Ar-H × 2), 4.89 (d, J = 8.8 Hz, 1H, -NH-), 4.57 (q, J = 6.8 Hz, 1H, -OCOCH-), 4.48 (dd, J = 11.4, 4.5 Hz, 1H, -COOCH-), 3.71 (dd, J = 9.9, 5.5 Hz, 1H, -OCH-), 3.13 (dd, J = 13.9, 5.9 Hz, 1H, PhCH_a_-), 3.02 (dd, J = 13.7, 6.9 Hz, 1H, PhCH_b_-), 2.95 (d, J = 9.3 Hz, 1H, -COCH-), 2.54 (td, J = 9.8, 4.1 Hz, 1H, -COCHCHCH_2_-), 2.22–2.18 (m, 2H), 1.95–0.79 (m, 18H), 1.39 (s, 9H, -C(CH_3_)_3_), 1.19 (s, 3H, -CH_3_), 1.19 (s, 3H, -CH_3_), 1.09 (s, 3H, -CH_3_), 1.04 (s, 3H, -CH_3_), 0.95 (s, 3H, -CH_3_), 0.82 (s, 3H, -CH_3_), 0.79 (s, 3H, -CH_3_), 0.75 (s, 3H, -CH_3_). ^13^C NMR (100 MHz, CDCl_3_) δ 210.9, 171.8, 155.1, 136.1, 129.3 (2C), 128.5 (2C), 126.9, 87.6, 85.3, 81.9, 79.8, 70.3, 57.2, 55.9, 55.8, 54.6, 54.2, 43.0, 40.4, 39.6, 39.6, 38.2, 37.9, 37.5, 36.5, 34.2, 31.9, 28.3 (3C), 27.9, 27.7, 26.6, 26.2, 24.8, 23.9, 23.3, 18.2, 16.6, 16.4, 16.0, 15.6. HRMS (ESI) *m*/*z* calcd C_44_H_67_NO_7_Na^+^ [M + Na]^+^ 744.4810, found 744.4781.

Compound **9d**, yield: 89%. ${\left[\alpha \right]}_{D}^{21}$ + 24.8 (*c* 1.00, CHCl_3_); ^1^H NMR (400 MHz, CDCl_3_) δ 7.33–7.24 (m, 5H, Ar-H × 5), 5.27 (d, J = 9.6 Hz, 1H, -NH-), 4.55 (d, J = 11.0 Hz, 1H, PhCH_a_-), 4.53 (dd, J = 11.3, 4.7 Hz, 1H, -COOCH-), 4.38 (d, J = 11.5 Hz, 1H, PhCH_b_-), 4.30 (dd, J = 9.8, 2.1 Hz, 1H, -OCOCH-), 4.21 (qd, J = 6.1, 1.9 Hz, 1H, PhCH_2_OCH-), 3.71 (dd, J = 9.8, 5.6 Hz, 1H, -OCH-), 2.96 (d, J = 9.3 Hz, 1H, -COCH-), 2.54 (td, J = 9.8, 4.1 Hz, 1H, -COCHCHCH_2_-), 2.21–2.19 (m, 2H), 2.09–2.08 (m, 1H), 1.93–0.82 (m, 17H), 1.45 (s, 9H, -C(CH_3_)_3_), 1.29 (d, J = 6.0 Hz, 3H, -CHCH_3_), 1.20 (s, 3H, -CH_3_), 1.19 (s, 3H, -CH_3_), 1.09 (s, 3H, -CH_3_), 1.04 (s, 3H, -CH_3_), 0.95 (s, 3H, -CH_3_), 0.87 (s, 3H, -CH_3_), 0.82 (s, 3H, -CH_3_), 0.75 (s, 3H, -CH_3_). ^13^C NMR (100 MHz, CDCl_3_) δ 210.9, 170.9, 156.0, 137.9, 128.2 (2C), 127.6, 127.4 (2C), 87.5, 85.2, 81.7, 79.7, 75.4, 70.8, 70.2, 58.6, 57.2, 55.8, 55.7, 54.2, 43.0, 40.4, 39.6, 38.2, 37.9, 37.5, 36.5, 34.2, 31.9, 28.3 (3C), 27.9, 27.7, 26.6, 26.2, 24.8, 23.9, 23.4, 18.2, 16.5 (2C), 16.4, 16.0, 15.6. HRMS (ESI) *m*/*z* calcd C_46_H_71_NO_8_Na^+^ [M + Na]^+^ 788.5072, found 788.5043.

Compound **9e**, yield: 90%. ${\left[\alpha \right]}_{D}^{24}$ + 38.6 (*c* 1.0, CHCl_3_); ^1^H NMR (400 MHz, CDCl_3_) δ 5.01 (s, 1H, -NH-), 4.55 (dd, J = 11.3, 5.2 Hz, 1H, -COOCH-), 3.90 (d, J = 5.2 Hz, 2H, -OCOCH_2_-), 3.71 (dd, J = 9.8, 5.6 Hz, 1H, -OCH-), 2.96 (d, J = 9.3 Hz, 1H, -COCH-), 2.54 (td, J = 9.8, 4.1 Hz, 1H, -COCHCHCH_2_-), 2.26–2.20 (m, 2H), 1.95–1.08 (m, 18H), 1.45 (s, 9H, -C(CH_3_)_3_), 1.20 (s, 3H, -CH_3_), 1.19 (s, 3H, -CH_3_), 1.10 (s, 3H, -CH_3_), 1.04 (s, 3H, -CH_3_), 0.97 (s, 3H, -CH_3_), 0.88 (s, 3H, -CH_3_), 0.87 (s, 3H, -CH_3_), 0.75 (s, 3H, -CH_3_); ^13^C NMR (100 MHz, CDCl_3_) δ 210.9, 170.0, 155.6, 87.6, 85.3, 81.7, 79.9, 70.3, 57.2, 55.9, 55.8, 54.2, 43.0, 42.6, 40.4, 39.6, 38.2, 38.0, 37.5, 36.5, 34.2, 31.9, 28.3 (3C), 28.0, 27.7, 26.7, 26.2, 24.8, 23.9, 23.4, 18.2, 16.6, 16.4, 16.1, 15.6. HRMS (ESI) *m*/*z* calcd C_37_H_61_NO_7_Na^+^ [M + Na]^+^ 654.4340, found 654.4316.

Compound **9f**, yield: 89%. ${\left[\alpha \right]}_{D}^{26}$ + 28.6(*c* 1.0, CHCl_3_); ^1^H NMR (400 MHz, CDCl_3_) δ 5.53 (d, J = 9.1 Hz, 1H, -NH-), 4.53–4.47 (m, 2H), 3.71 (dd, J = 9.9, 5.5 Hz, 1H, -OCH-), 2.96 (d, J = 9.3 Hz, 1H, -COCH-), 2.92 (dd, J = 17.0, 4.4 Hz, 1H, -CH_a_COO*t*Bu), 2.73 (dd, J = 17.0, 4.4 Hz, 1H, -CH_b_COO*t*Bu), 2.54 (td, J = 10.0, 4.0 Hz, 1H, -COCHCHCH_2_-), 2.22–2.18 (m, 2H), 1.93–0.82 (m, 18H), 1.45 (s, 9H, -C(CH_3_)_3_), 1.43 (s, 9H, -C(CH_3_)_3_), 1.20 (s, 3H, -CH_3_), 1.19 (s, 3H, -CH_3_), 1.09 (s, 3H, -CH_3_), 1.04 (s, 3H, -CH_3_), 0.97 (s, 3H, -CH_3_), 0.88 (s, 3H, -CH_3_), 0.86 (s, 3H, -CH_3_), 0.75 (s, 3H, -CH_3_). ^13^C NMR (100 MHz, CDCl_3_) δ 210.9, 170.9, 170.3, 155.5, 87.6, 85.3, 82.0, 81.6, 79.8, 70.3, 57.2, 55.9, 55.8, 54.2, 50.2, 43.0, 40.4, 39.7, 38.2, 38.0, 37.7, 37.5, 36.6, 34.2, 31.9, 28.3 (3C), 28.1 (3C), 28.0, 27.7, 26.6, 26.2, 24.8, 23.9, 23.3, 18.2, 16.6, 16.5, 16.1, 15.6. HRMS (ESI) *m*/*z* calcd C_43_H_71_NO_9_Na^+^ [M + Na]^+^ 768.5021, found 768.4992.

Compound **10a**, yield: 91%. ${\left[\alpha \right]}_{D}^{20}$ + 35.9 (*c* 1.00, CH_3_OH); ^1^H NMR (400 MHz, CD_3_OD) δ 4.65 (dd, J = 10.4, 6.0 Hz, 1H, -COOCH-), 4.21 (dd, J = 7.0, 5.6 Hz, 1H, -OCOCH-), 3.70 (dd, J = 8.9, 6.7 Hz, 1H, -OCH-), 3.07 (d, J = 9.6 Hz, 1H, -COCH-), 2.67 (t, J = 7.4 Hz, 2H, -SCH_2_-), 2.50 (td, J = 9.7, 4.4 Hz, 1H, -COCHCHCH_2_-), 2.38 (t, J = 13.2 Hz, 1H, -COCH_a_CH-), 2.31–2.22 (m, 1H), 2.16–2.09 (m, 3H), 2.11 (s, 3H, -SCH_3_), 1.93–0.97 (m, 17H), 1.26 (s, 3H, -CH_3_), 1.14 (s, 3H, -CH_3_), 1.10 (s, 3H, -CH_3_), 1.03 (s, 3H, -CH_3_), 1.03 (s, 3H, -CH_3_), 0.95 (s, 3H, -CH_3_), 0.93 (s, 3H, -CH_3_), 0.77 (s, 3H, -CH_3_). ^13^C NMR (100 MHz, CD_3_OD) δ 214.1, 170.3, 88.8, 86.6, 85.0, 72.0, 58.4, 57.3, 56.9, 55.8, 53.0, 44.3, 41.7, 40.6, 39.2, 39.1, 38.7, 37.0, 35.3, 33.0, 30.9, 30.2, 28.6, 27.4, 26.9, 26.2, 25.9, 25.6, 24.4, 19.3, 17.1 (2C), 16.5, 16.1, 14.9. HRMS (ESI) *m*/*z* calcd C_35_H_60_NO_5_S^+^ [M + H]^+^ 606.4187, found 606.4164.

Compound **10b**, yield: 92%. ${\left[\alpha \right]}_{D}^{25}$ + 24.4(*c* 1.0, CHCl_3_); ^1^H NMR (400 MHz, CDCl_3_) δ 4.58 (dd, J = 10.6, 3.2 Hz, 1H, -COOCH-), 3.81 (br, 1H), 3.71 (dd, J = 9.6, 5.5 Hz, 1H, -COCH-), 3.66 (d, J = 6.3 Hz, 1H, -OCOCH-), 3.60 (br, 1H), 2.96 (d, J = 9.3 Hz, 1H, -COCH-), 2.54 (td, J = 9.8, 3.8 Hz, 1H, -COCHCHCH_2_-), 2.38–2.16 (m, 3H), 1.92–0.81 (m, 24H), 1.21 (s, 3H, -CH_3_), 1.19 (s, 3H, -CH_3_), 1.10 (s, 3H, -CH_3_), 1.04 (s, 3H, -CH_3_), 0.96 (s, 3H, -CH_3_), 0.90 (s, 6H, -CH_3_ × 2), 0.76 (s, 3H, -CH_3_). ^13^C NMR (100 MHz, CDCl_3_) δ 211.0, 167.7, 87.6, 85.3, 83.7, 70.4, 57.2, 57.2, 55.9, 55.8, 54.2, 43.0, 40.4, 38.2, 38.1, 38.1, 37.8, 37.5, 36.4, 34.2, 31.9, 28.1, 27.7, 26.7, 26.7, 26.2, 24.9, 23.8, 18.2, 18.2, 17.3, 16.6 (2C), 16.0, 15.6. HRMS (ESI) *m*/*z* calcd C_35_H_59_NO_5_Na^+^ [M + Na]^+^ 596.4285, found 596.4266.

Compound **10c**, yield: 93%. ${\left[\alpha \right]}_{D}^{25}$ + 22.5(*c* 1.0, CHCl_3_); ^1^H NMR (400 MHz, CDCl_3_) δ 7.36–7.25 (m, 5H, Ar-H × 5), 4.47 (dd, J = 10.2, 3.8 Hz, 1H, -COOCH-), 4.21 (dd, J = 11.3, 5.5 Hz, 1H, -OCOCH-), 3.70 (dd, J = 9.3, 5.8 Hz, 1H, -COCH-), 3.31–3.23 (m, 2H), 2.95 (d, J = 9.3 Hz, 1H, -COCH-), 2.85–2.85 (m, 1H), 2.53 (td, J = 9.6, 4.0 Hz, 1H, -COCHCHCH_2_-), 2.32–2.14 (m, 2H), 1.94–0.75 (m, 17H), 1.19 (s, 3H, -CH_3_), 1.18 (s, 3H, -CH_3_), 1.09 (s, 3H, -CH_3_), 1.05 (s, 3H, -CH_3_), 0.92 (s, 3H, -CH_3_), 0.75 (s, 3H, -CH_3_), 0.74 (s, 3H, -CH_3_), 0.71 (s, 3H, -CH_3_). ^13^C NMR (100 MHz, CDCl_3_) δ 211.0, 168.9, 133.7, 129.3 (2C), 129.1 (2C), 127.9, 87.5, 85.3, 84.1, 70.4, 57.2, 55.9, 55.7, 54.3, 54.2, 43.0, 40.4, 39.6, 38.0, 37.7, 37.4, 36.5, 36.4, 34.2, 31.9, 27.8, 27.6, 26.9, 26.7, 26.2, 24.9, 23.8, 18.1, 16.6, 16.2, 16.0, 15.6. HRMS (ESI) *m*/*z* calcd C_39_H_59_NO_5_Na^+^ [M + Na]^+^ 644.4285, found 644.4265.

Compound **10d**, yield: 81%. ${\left[\alpha \right]}_{D}^{22}$ + 32.8 (*c* 0.40, CH_3_OH); ^1^H NMR (400 MHz, CD_3_OD) δ 7.37–7.26 (m, 5H, Ar-H × 5), 4.69 (d, J = 11.5 Hz, 1H, PhCH_a_-), 4.62 (dd, J = 10.7, 4.9 Hz, 1H, -COOCH-), 4.46 (d, J = 11.5 Hz, 1H, PhCH_b_-), 4.29 (qd, J = 6.5, 3.0 Hz, 1H, PhCH_2_OCH-), 4.13 (d, J = 3.0 Hz, 1H, -OCOCH-), 3.70 (dd, J = 8.8, 6.6 Hz, 1H, -COCH-), 3.06 (d, J = 9.3 Hz, 1H, -COCH-), 2.50 (td, J = 9.6, 4.4 Hz, 1H, -COCHCHCH_2_-), 2.37 (t, J = 13.2 Hz, 1H, -COCH_a_CH-), 2.13 (dd, J = 12.9, 3.6 Hz, 1H, -COCH_b_CH-), 1.93–0.91 (m, 18H), 1.43 (d, J = 6.6 Hz, 3H, -CHCH_3_), 1.25 (s, 3H, -CH_3_), 1.14 (s, 3H, -CH_3_), 1.10 (s, 3H, -CH_3_), 1.03 (s, 3H, -CH_3_), 1.01 (s, 3H, -CH_3_), 0.93 (s, 3H, -CH_3_), 0.82 (s, 3H, -CH_3_), 0.76 (s, 3H, -CH_3_). ^13^C NMR (100 MHz, CD_3_OD) δ 214.1, 168.8, 138.8, 129.4 (2C), 128.9, 128.8 (2C), 88.8, 86.6, 85.0, 74.0, 72.0, 72.0, 59.2, 58.4, 57.3, 56.9, 55.8, 44.3, 41.7, 40.6, 39.2, 39.0, 38.7, 37.0, 35.3, 33.0, 28.7, 27.4, 26.9, 26.2, 25.9, 25.6, 24.4, 19.3, 17.1, 17.0, 16.5, 16.4, 16.1. HRMS (ESI) *m*/*z* calcd C_41_H_64_NO_6_^+^ [M + H]^+^ 666.4728, found 666.4702.

Compound **10e**, yield: 93%. ${\left[\alpha \right]}_{D}^{26}$ + 18.7(*c* 1.0, CHCl_3_); ^1^H NMR (400 MHz, CD_3_OD) δ 4.65 (dd, J = 11.0, 5.5 Hz, 1H, -COOCH-), 3.86 (d, J = 17.3 Hz, 1H, -OCOCH_a_-), 3.80 (d, J = 17.0 Hz, 1H, -OCOCH_b_-), 3.70 (dd, J = 8.8, 6.6 Hz, 1H, -COCH-), 3.07 (d, J = 9.6 Hz, 1H, -COCH-), 2.50 (td, J = 9.8, 4.6 Hz, 1H, -COCHCHCH_2_-), 2.38 (t, J = 13.3 Hz, 1H, -COCH_a_CH-), 2.13 (dd, J = 13.1, 3.7 Hz, 1H, -COCH_b_CH-), 1.93–0.87 (m, 18H), 1.26 (s, 3H, -CH_3_), 1.14 (s, 3H, -CH_3_), 1.10 (s, 3H, -CH_3_), 1.03 (s, 3H, -CH_3_), 1.02 (s, 3H, -CH_3_), 0.93 (s, 3H, -CH_3_), 0.92 (s, 3H, -CH_3_), 0.77 (s, 3H, -CH_3_). ^13^C NMR (100 MHz, CD_3_OD) δ 214.1, 168.5, 88.9, 86.6, 84.5, 72.0, 58.5, 57.3, 57.0, 55.9, 44.3, 41.8, 41.1, 40.6, 39.3, 39.1, 38.7, 37.0, 35.3, 33.0, 28.4, 27.4, 26.9, 26.2, 25.9, 25.6, 24.5, 19.3, 17.1, 16.8, 16.6, 16.1. HRMS (ESI) *m*/*z* calcd C_32_H_53_NO_5_Na^+^ [M + Na]^+^ 554.3816, found 554.3797.

Compound **10f**, yield: 90%. ${\left[\alpha \right]}_{D}^{30}$ + 21.9(*c* 1.0, CHCl_3_); ^1^H NMR (400 MHz, CD_3_OD) δ 4.63 (dd, J = 10.7, 5.5 Hz, 1H, -COOCH-), 4.28–4.25 (m, 1H, -OCOCH-), 3.70 (dd, J = 8.7, 6.7 Hz, 1H, -COCH-), 3.07 (d, J = 9.6 Hz, 1H, -COCH-), 3.02–2.89 (m, 2H), 2.50 (td, J = 9.3, 4.5 Hz, 1H, -COCHCHCH_2_-), 2.37 (t, J = 13.2 Hz, 1H, -COCH_a_CH-), 2.13 (dd, J = 13.2, 3.6 Hz, 1H, -COCH_b_CH-), 1.93–0.87 (m, 18H), 1.28 (s, 3H, -CH_3_), 1.14 (s, 3H, -CH_3_), 1.10 (s, 3H, -CH_3_), 1.03 (s, 3H, -CH_3_), 1.02 (s, 3H, -CH_3_), 0.93 (s, 3H, -CH_3_), 0.90 (s, 3H, -CH_3_), 0.77 (s, 3H, -CH_3_). ^13^C NMR (100 MHz, CD_3_OD) δ 214.1, 169.5, 169.3, 88.8, 86.6, 84.8, 72.0, 58.4, 57.3, 57.0, 55.8, 51.1, 44.3, 41.7, 40.6, 39.2, 39.1, 38.7, 37.0, 35.3, 33.0, 31.7, 28.5, 27.4, 26.9, 26.2, 25.9, 25.6, 24.4, 19.3, 17.1, 16.9, 16.5, 16.1. HRMS (ESI) *m*/*z* calcd C_34_H_55_NO_7_Na^+^ [M + Na]^+^ 612.3871, found 612.3847.

### 3.2. Cell Culture and NO Generation Assay

RAW264.7 cells were maintained in RPMI-1640 medium (containing 10% FBS) under a 5% CO_2_ humidified condition at 37 °C and cellular NO release of treatment cells was evaluated using the Griess assay. Briefly, after pretreatment with the test compounds for 2 h, the experimental cells were treated with LPS (1 μg/mL) for another 24 h. Beyotime Griess reagent (China) was then used to assess NO levels in culture supernatants using a SpectraMax-M3 microplate reader (540 nm) as previously described [40].

### 3.3. Cell Viability

Cell survival rate was assessed in parallel with the NO generation assay using the MTT method as previously described [26]. Briefly, the compound-treated cells were further incubated with MTT reagent for 4 h. Subsequently, the resultant precipitates were dissolved in 150 μL DMSO and the OD was detected at 570 nm.

### 3.4. ELISAs

The experimental cells (0.5 × 10^4^ per well) were cultured for 24 h, pretreated with **5c** or HSS for 2 h and cotreated with 1 μg/mL LPS for another 6 h (for TNF-α) or 24 h (for IL-1β). The supernatant cytokine levels were next assessed using ELISA kits (Elabscience, Wuhan, China) based on the manufacturer’s instructions [41].

### 3.5. Western Blotting

Target protein levels were estimated using Western blotting as published elsewhere [42,43]. Briefly, total cellular proteins of compound-treated cells were separated on the SDS-PAGE and blotted to the PVDF membrane (Millipore), then treated with Beyotime primary antibody (anti-iNOS (AF7281), anti-p-iκB (AF1870), anti-iκB (AF1282), anti-p-p65 (AN371), anti-p65 (AF1234), anti-p-ERK1/2 (AF1891), anti-ERK1/2 (AF1315), anti-p-SAPK/JNK (AF1762), anti-SAPK/JNK (AJ518), et al.) and Beyotime HRP-conjugated secondary antibody. The proteins were analyzed using chemiluminescence with the Beyotime ECL reagent.

### 3.6. Cellular Thermal Shift Assay (CETSA)

Protein–compound interactions in physiological environments were analyzed using CETSA [38,39]. RAW264.7 cells (2 × 10^7^ cells/mL) were seeded for 24 h. After 2 h treatment with **5c** (20 μM), HSS (20 μM), or DMSO, the cells were split into eight equal groups. The groups were heated for 3 min at 40, 43, 46, 49, 52, 55, 58, and 61 °C, followed by incubation at RT for 3 min. The cells then underwent five freeze–thaw cycles by placing in liquid nitrogen for 15 s followed by heating in a heating block at 30 °C for 3 min and brief vortexing. The lysates were centrifuged (12,000 rpm, 10 min) and the GR concentrations measured using Western blotting used GAPDH as the control.

### 3.7. Statistical Analyses

The data are represented as the means ± SD and, when compared using Student’s *t*-test, *p* < 0.05 values were statistically significant. All experiments were repeated in triplicate.

## 4. Conclusions

Pyxinol, the key pharmacophore of the liver metabolites of ginsenosides [12,13,14], was selected as the core skeleton for drug development in several groups. The effects of the dehydrogenation of pyxinol at C-12 on its potency have been rarely studied. In this study, 24 amino acid residue-conjugated 12-dehydropyinol derivatives were produced and their inhibiting effects on LPS-triggered NO production were assessed. Compared with Y13, which was identified to be the most potent derivative of pyxinol, half of the 12-dehydropyinol derivatives exhibited comparable potencies and several of them even showed much higher potencies than Y13. These results indicate that dehydrogenation at C-12 largely promotes the anti-inflammatory activity of C-3-modified derivatives. The SAR study further alluded that modification of 12-dehydropyxinol at C-3 with an *N*-Boc-protected aromatic amino acid led to an obvious increase in its anti-inflammatory activity, with 24*R* being the most preferred. Derivative **5c** was then identified as exhibiting the most potent activity and relatively low cytotoxicity. Further studies have indicated that **5c** exerted robust GR-independent anti-inflammatory activity by inhibiting the activation of NF-κB and MAPK to suppress IL-1β, TNF-α, and iNOS upregulation. The selective interaction of **5c** with JNK is the possible mechanism for inhibiting MAPK activation. These data highlight that 12-dehydropyinol derivatives hold immense drug-development potential owing to their anti-inflammatory activity.

## Data Availability

Not applicable.

## Supplementary Materials

The following supporting information can be downloaded at: https://www.mdpi.com/article/10.3390/molecules28031307/s1, ^1^H NMR and ^13^C NMR spectra of compounds.

## Sample Availability

Samples of the compounds are available from the authors.
