# Supplementary material for: Design, Synthesis, and Anti-Inflammatory Activities of 12-Dehydropyxinol Derivatives
Source: Molecules. 2023 Jan 30;28(3):1307. doi: 10.3390/molecules28031307 (PMC9921557; doi:10.3390/molecules28031307)

# Supporting information

# Design, synthesis, and anti-inflammatory activities of 12-dehydropyxinol derivatives

**Yunxiao Wang [1,†], Xiaoliang Mi [1,†], Yuan Du [1,†], Shuang Li [1], Liping Yu [1], Meng Gao [1], Xiaoyue Yang [1], Zhihua Song [1], Hui Yu [2,*], and Gangqiang Yang [1,*]**

[a]School of Pharmacy, Key Laboratory of Molecular Pharmacology and Drug Evaluation (Yantai University), Ministry of Education, Collaborative Innovation Center of Advanced Drug Delivery System and Biotech Drugs in Universities of Shandong, Yantai University, Yantai, 264005, China

[b]College of Food Engineering, Ludong University, Yantai, 264025, China

[†]The authors contributed equally.

*Corresponding authors：oceanygq@ytu.edu.cn (G.Y.), zoehuihui@hotmail.com (H.Y.)

Table of contents:

$^{1}$H and $^{13}$C NMR Spectra SI 2

## $^{1}$H and $^{13}$C NMR Spectra of **5a**

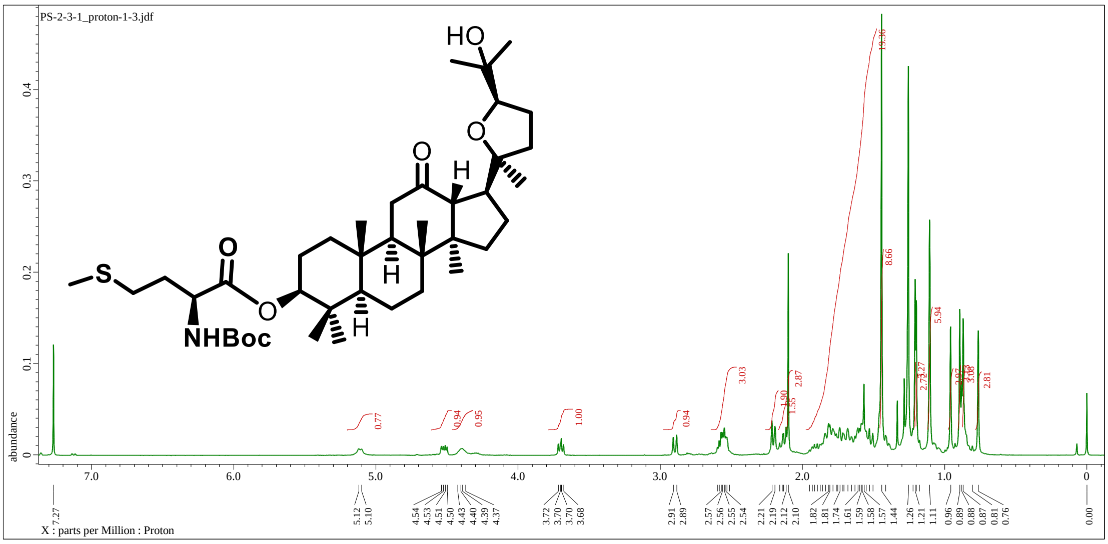

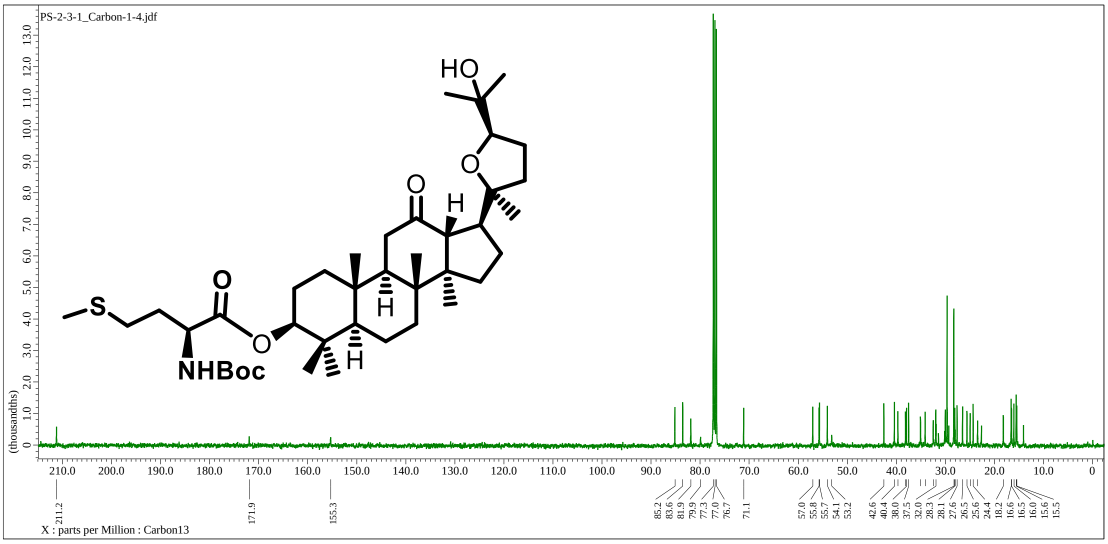

# $^1$H and $^{13}$C NMR Spectra of **5b**

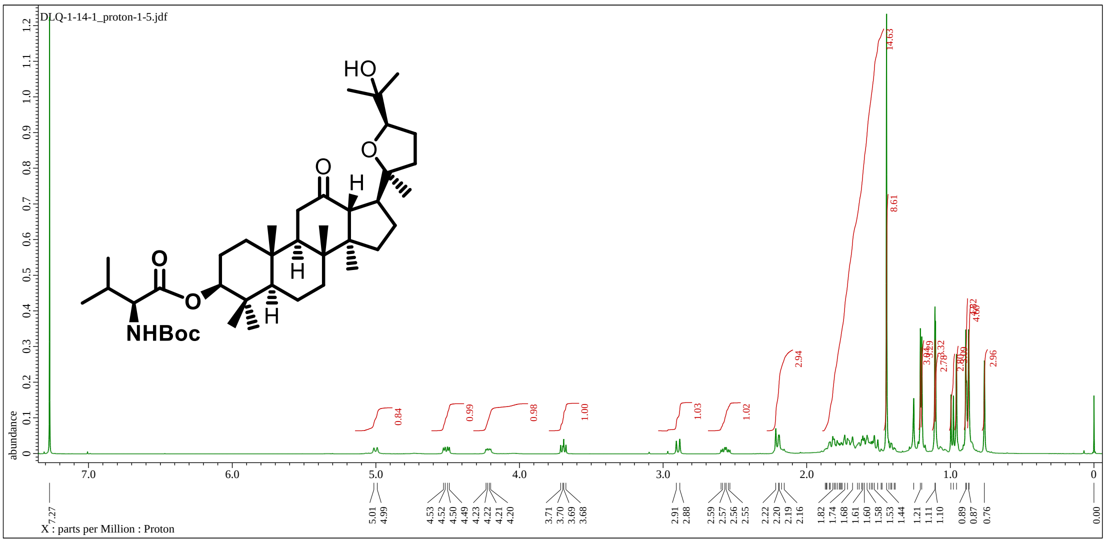


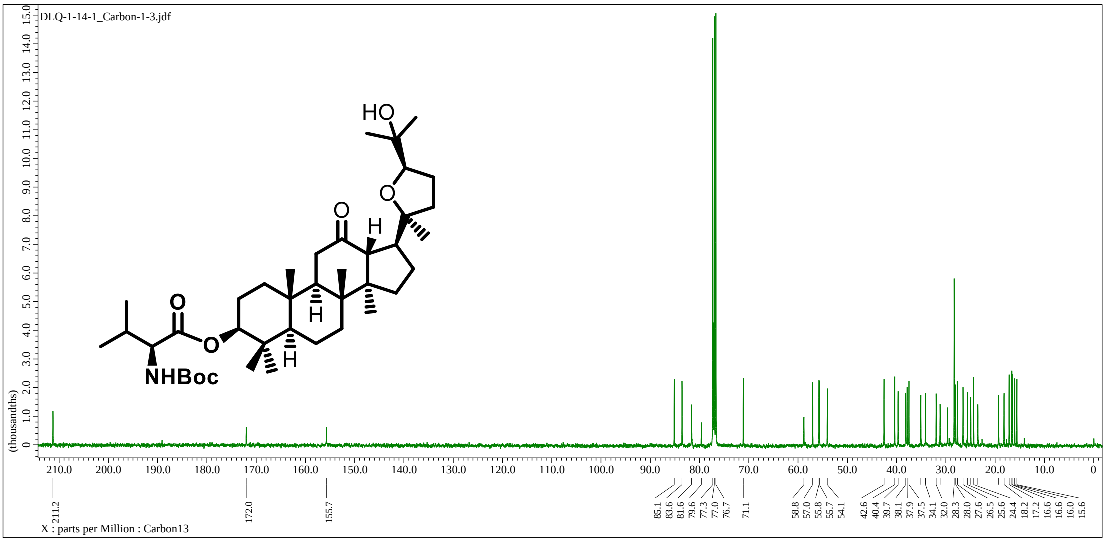

$^{1}$H and $^{13}$C NMR Spectra of **5c**

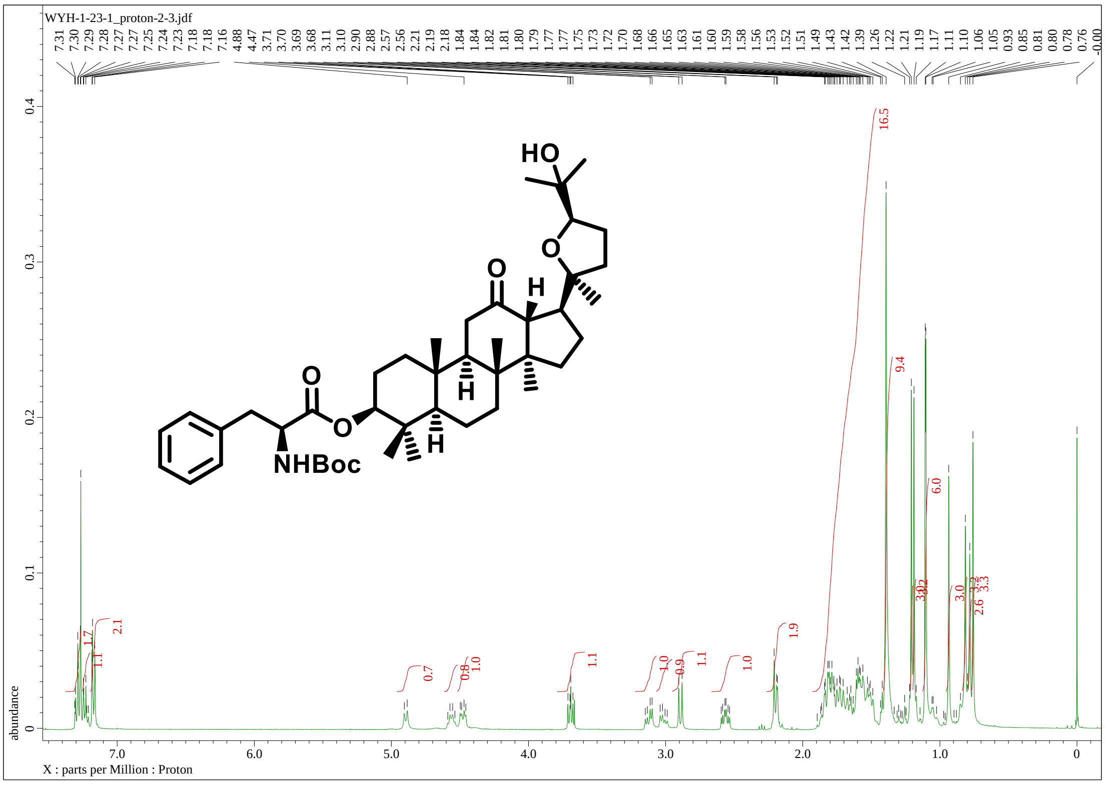

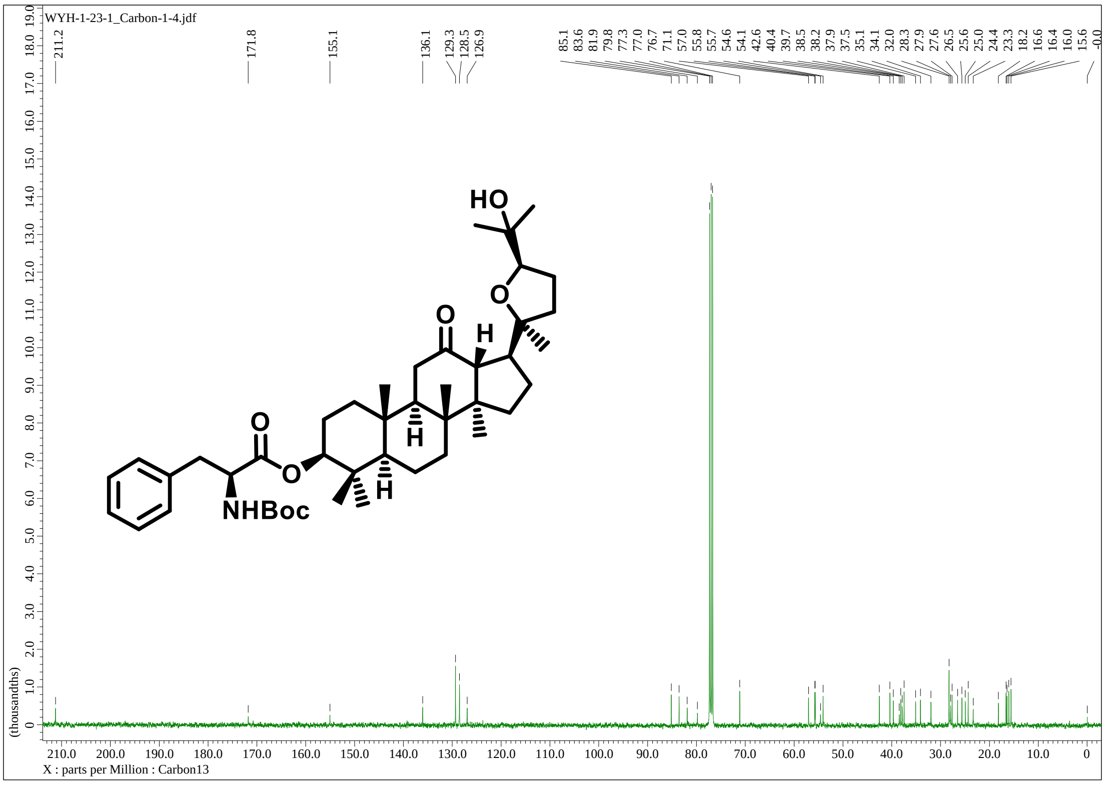

# $^{1}H$ and $^{13}C$ NMR Spectra of **5d**

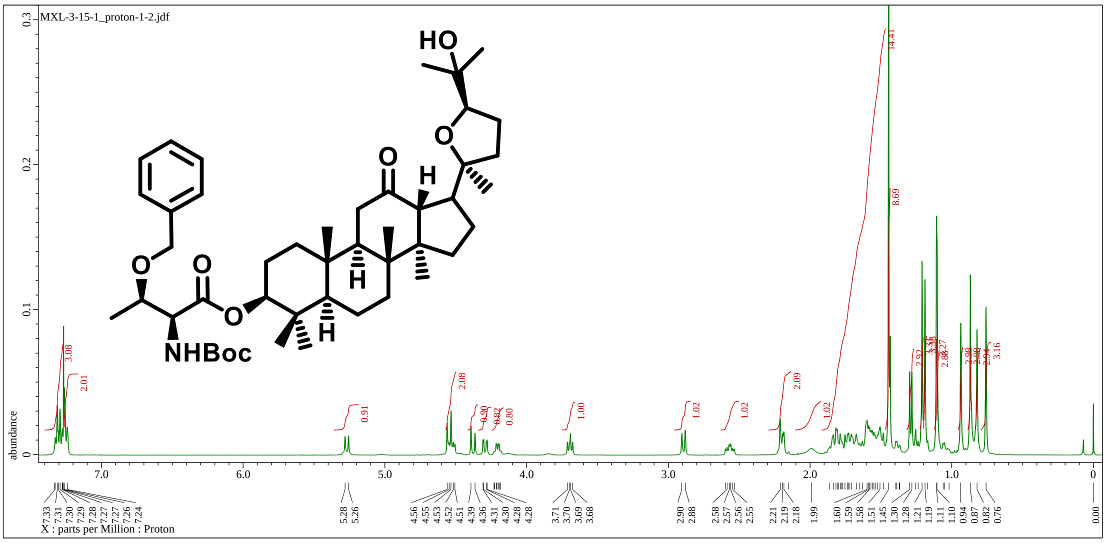


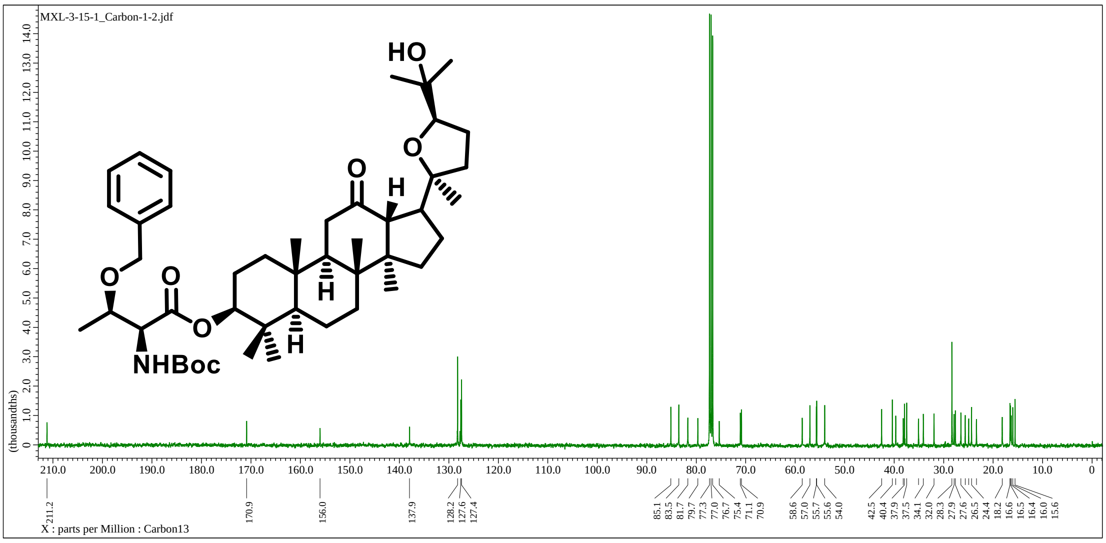

# $^{1}$H and $^{13}$C NMR Spectra of **5e**

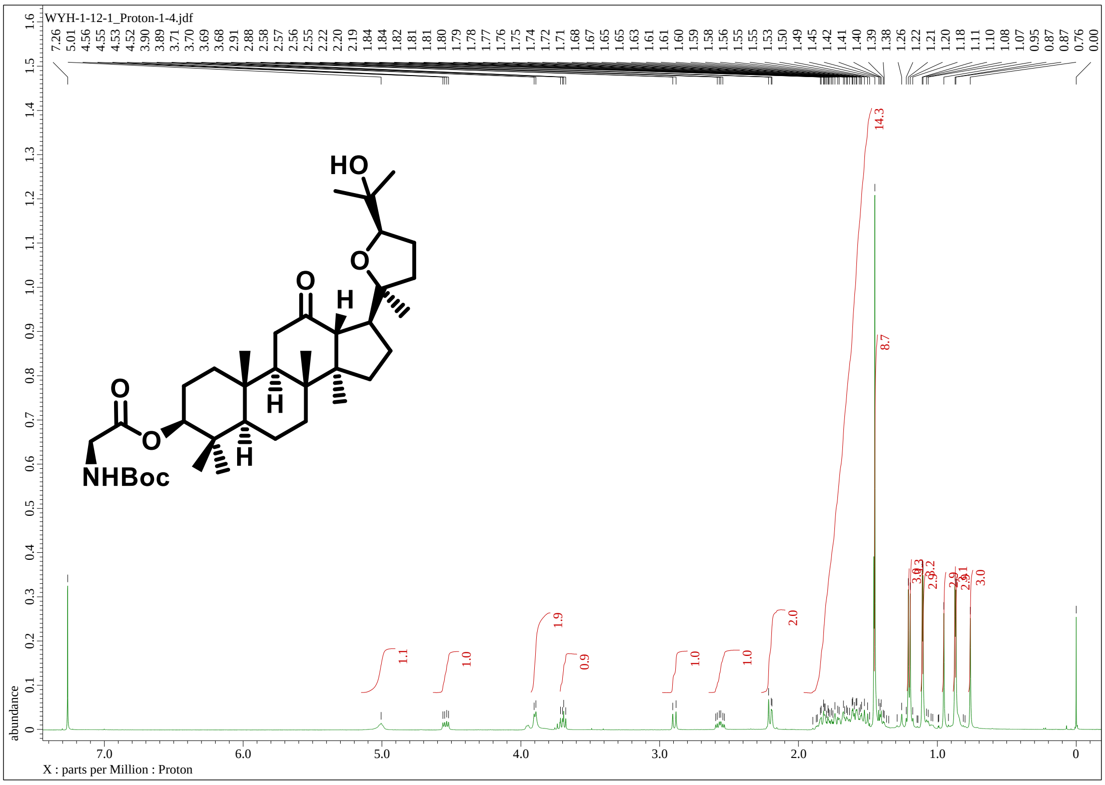


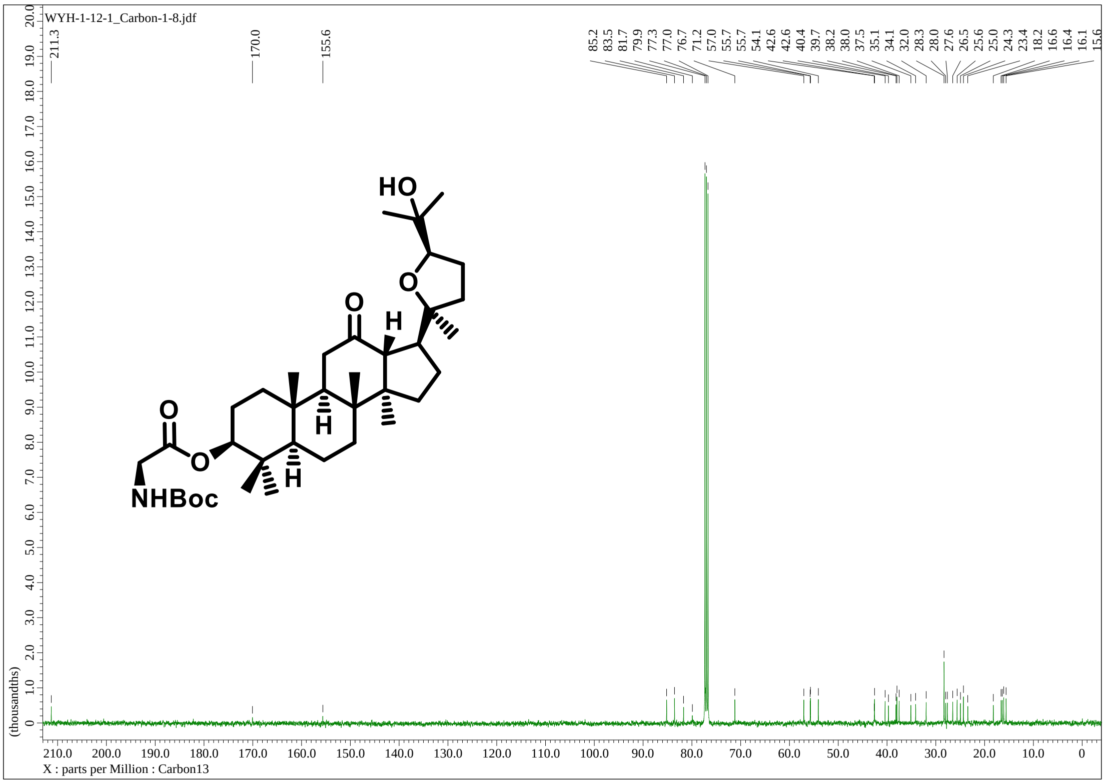

# $^{1}$H and $^{13}$C NMR Spectra of **5f**

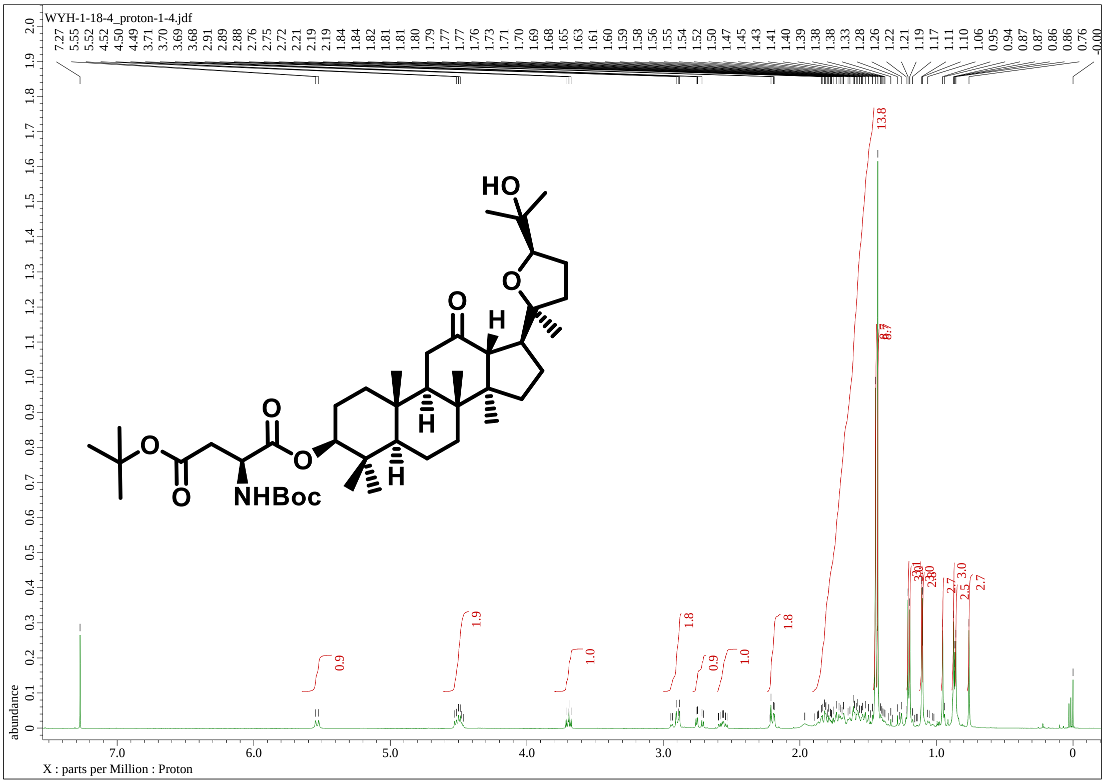


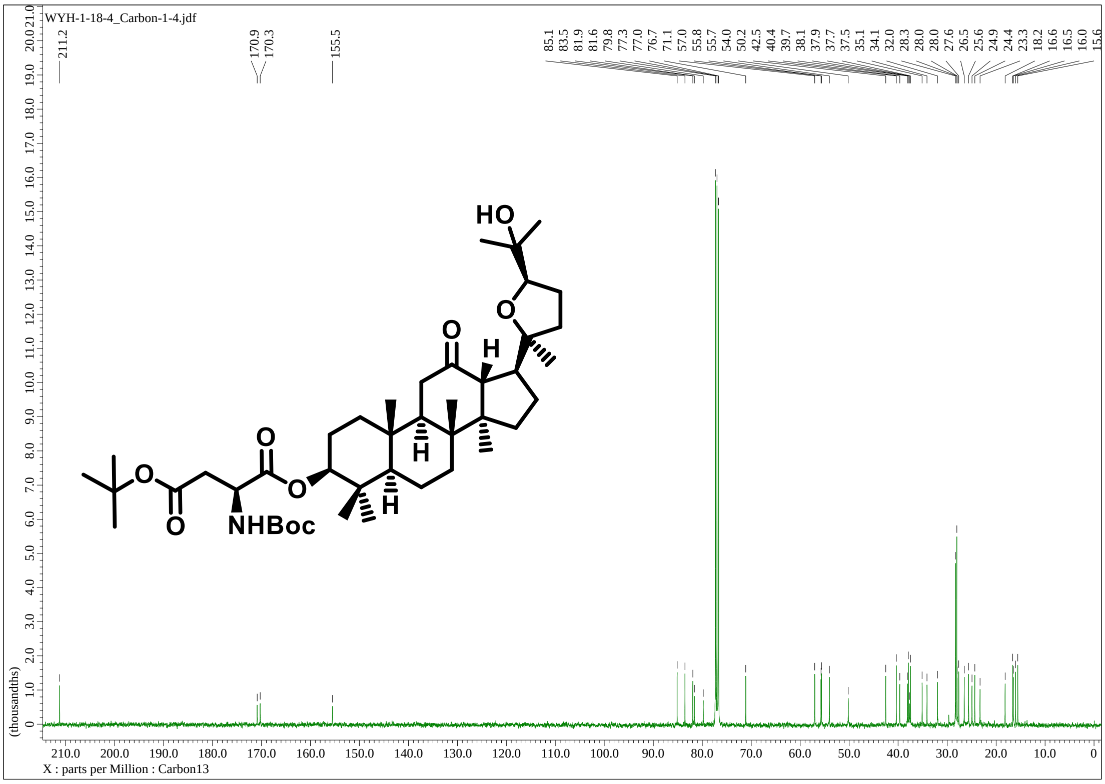

$^{1}H$ and $^{13}C$ NMR Spectra of **6a**

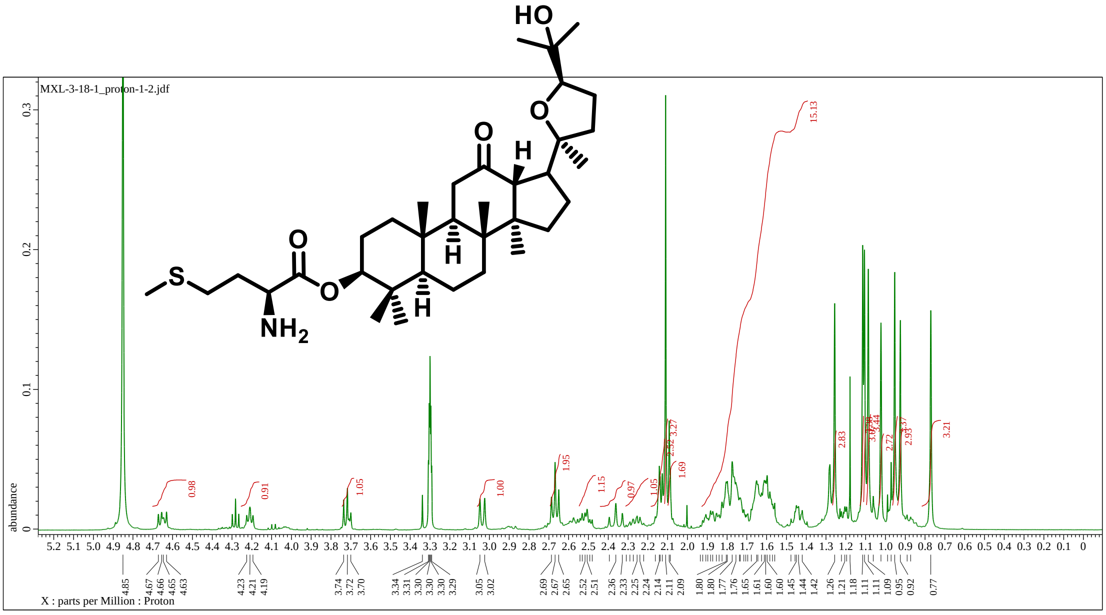


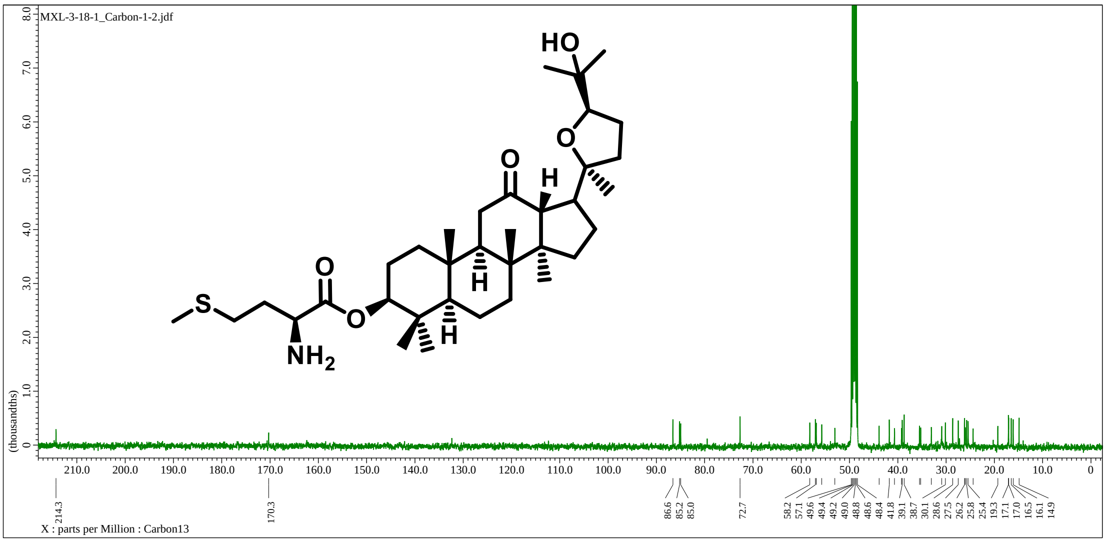

# $^{1}H$ and $^{13}C$ NMR Spectra of **6b**

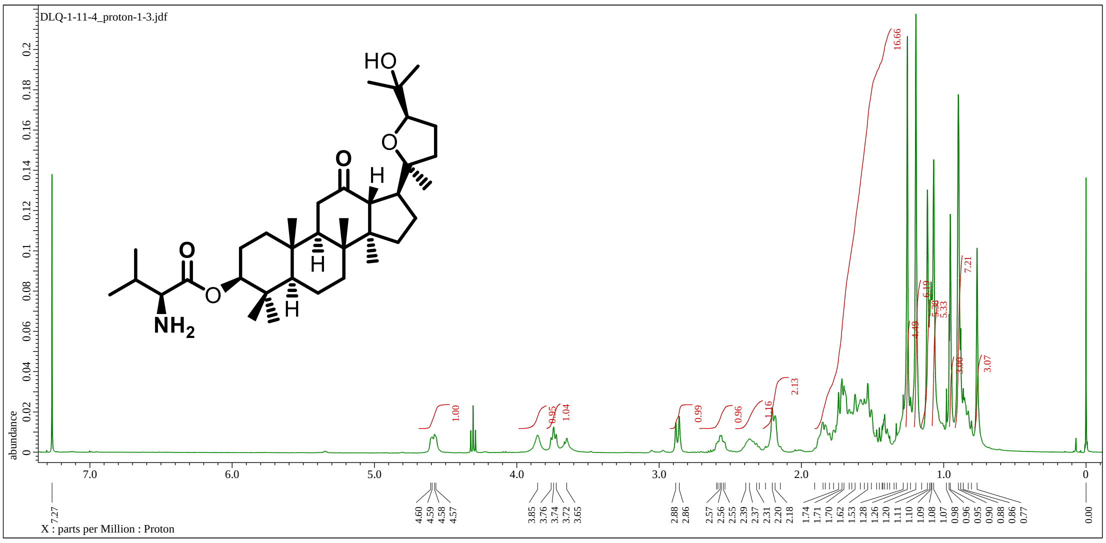


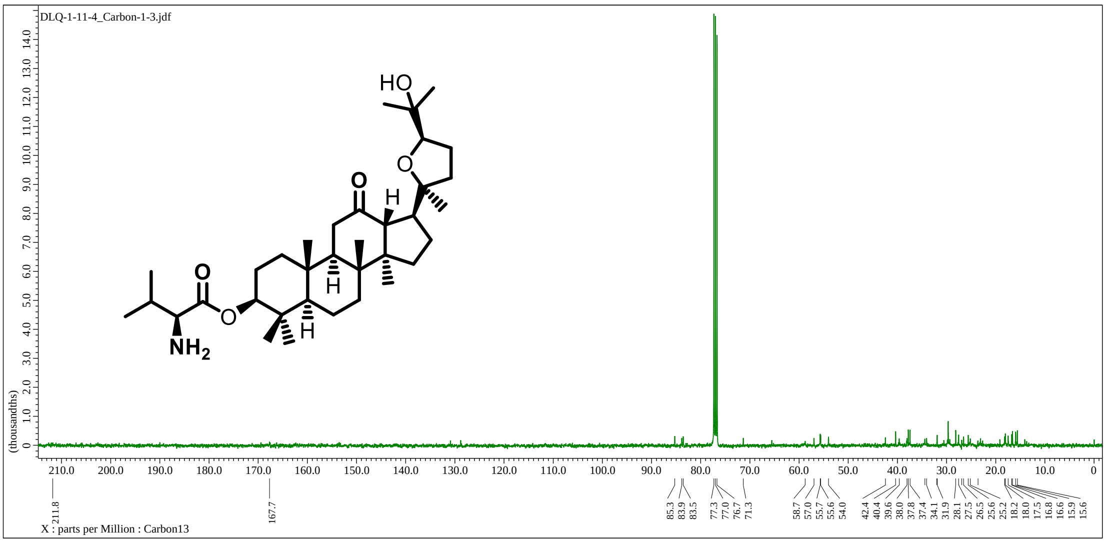

# $^{1}H$ and $^{13}C$ NMR Spectra of **6c**

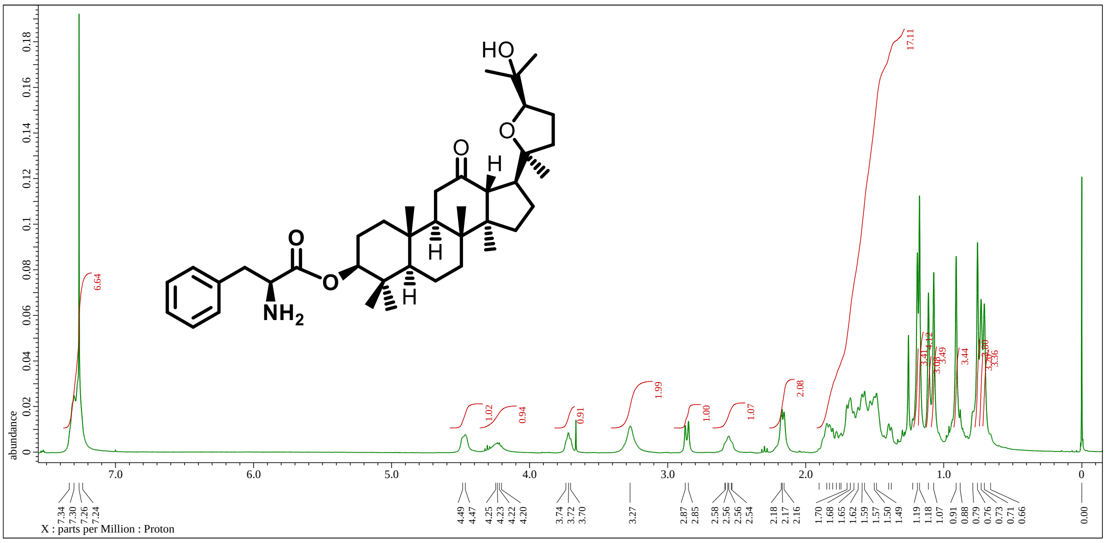


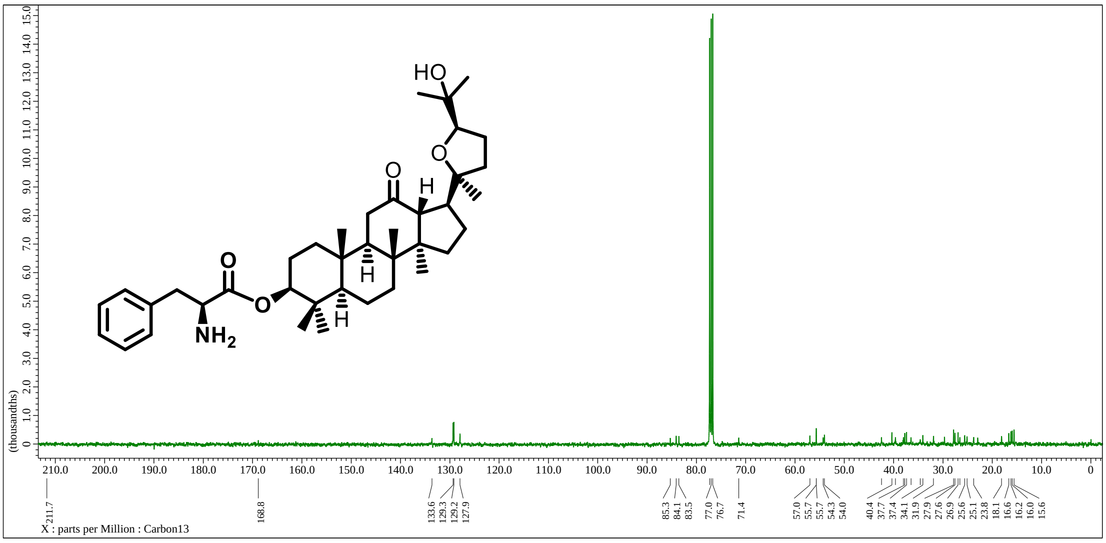

# $^1$H and $^{13}$C NMR Spectra of **6d**

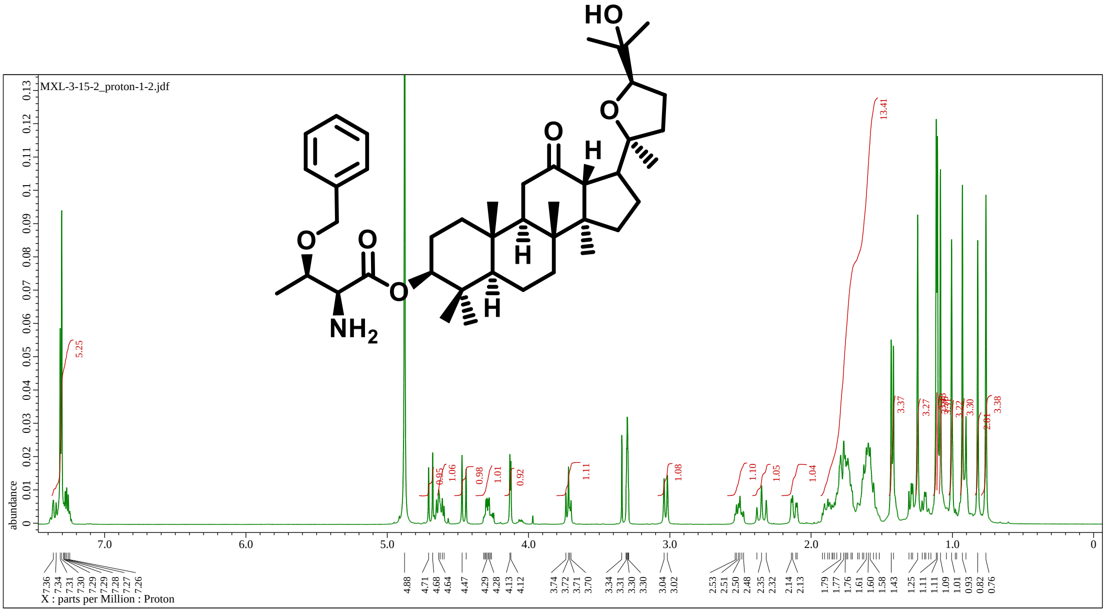


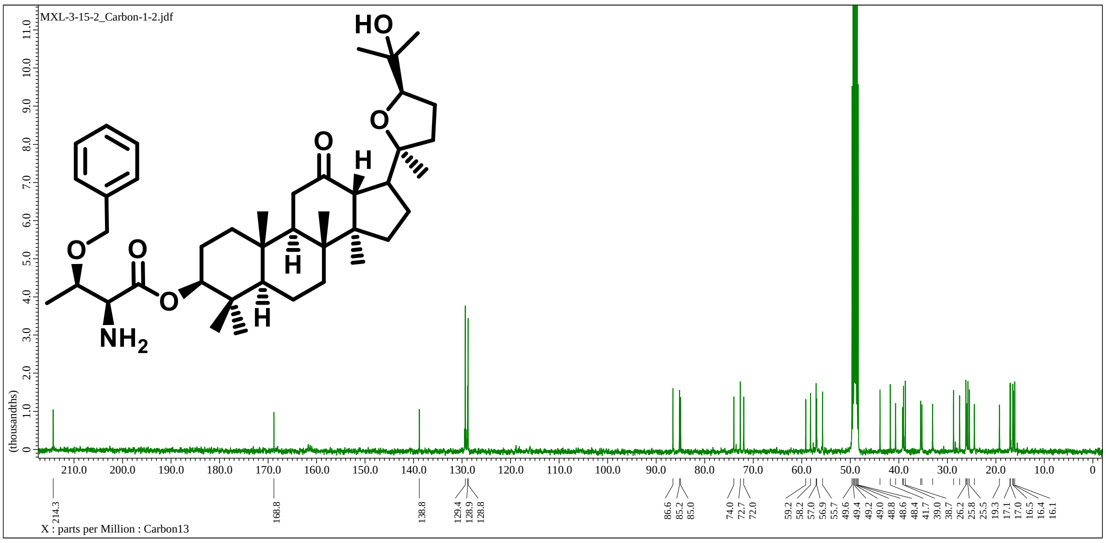

# $^{1}H$ and $^{13}C$ NMR Spectra of **6e**

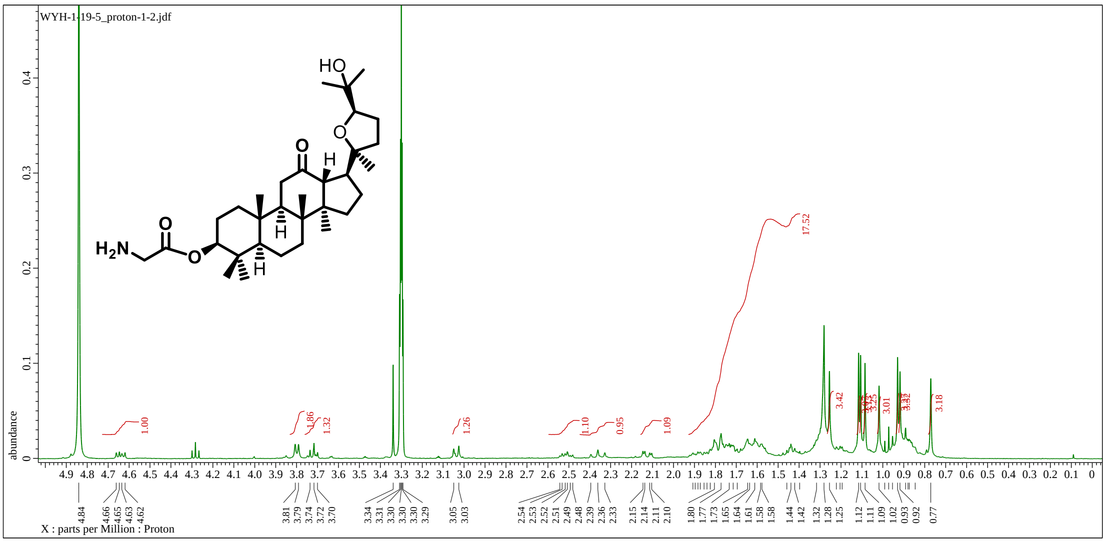

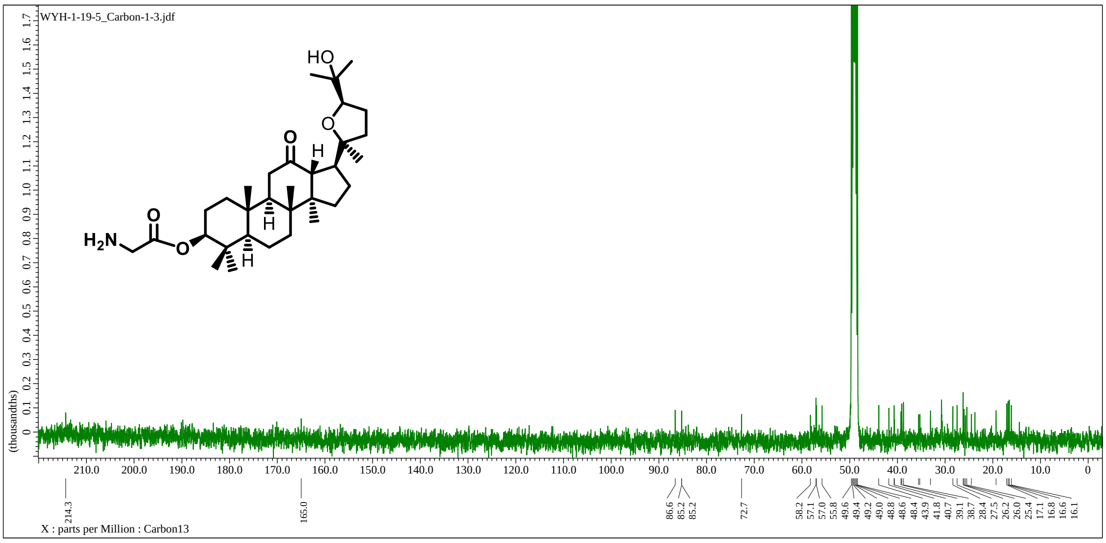

# $^{1}$H and $^{13}$C NMR Spectra of **6f**

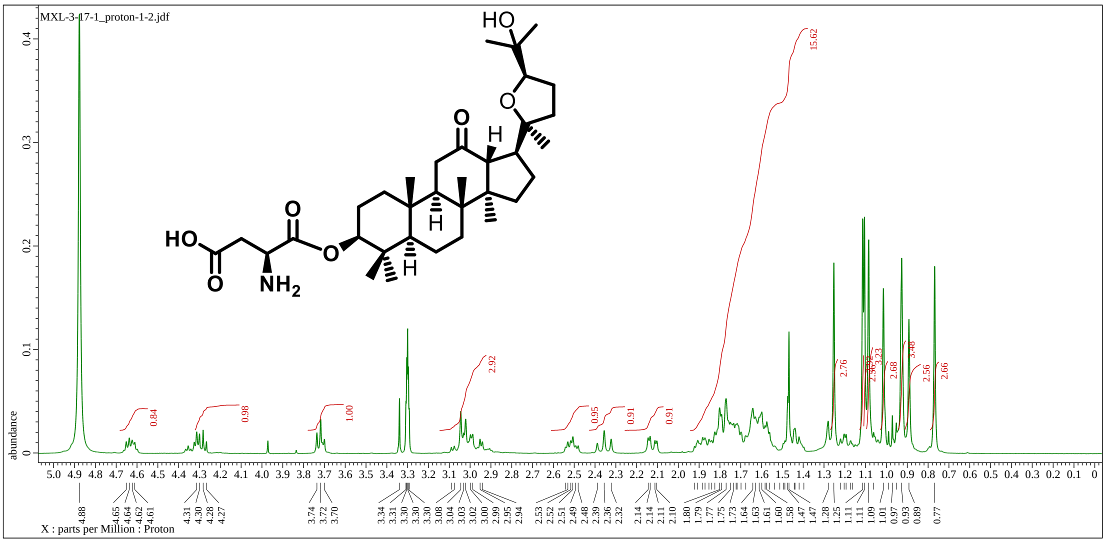


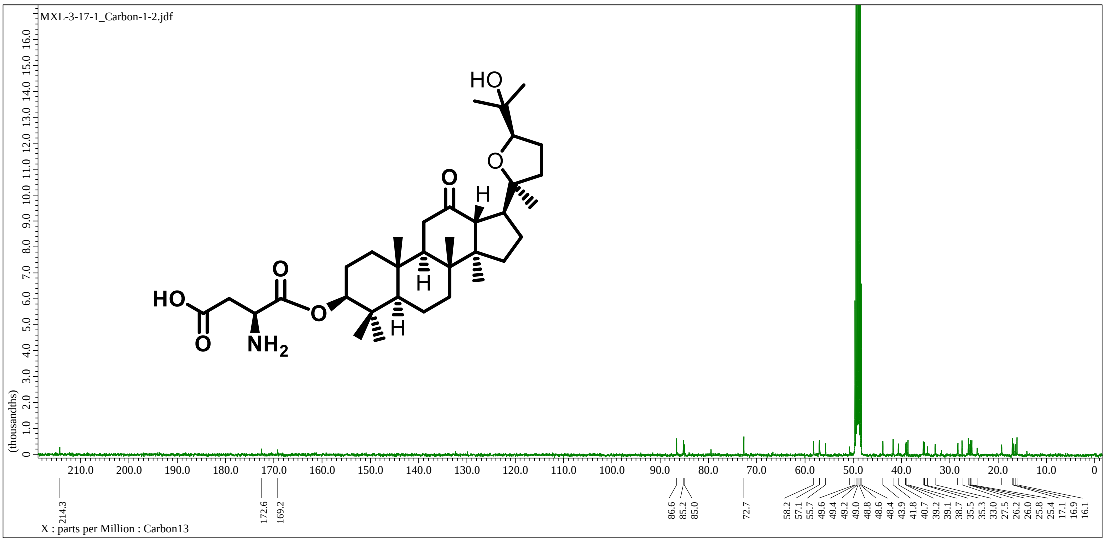

## $^{1}H$ and $^{13}C$ NMR Spectra of **9a**

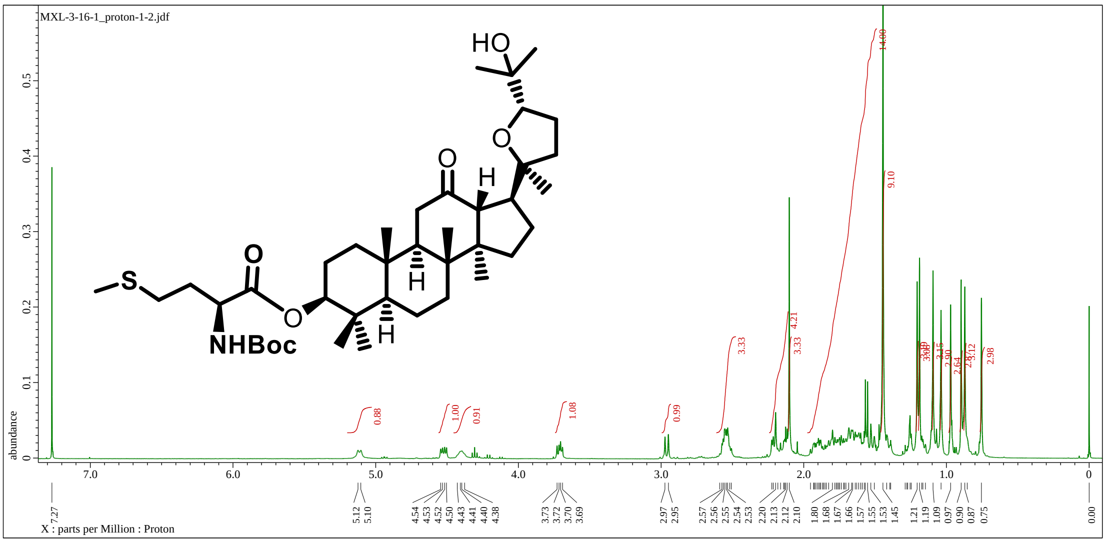

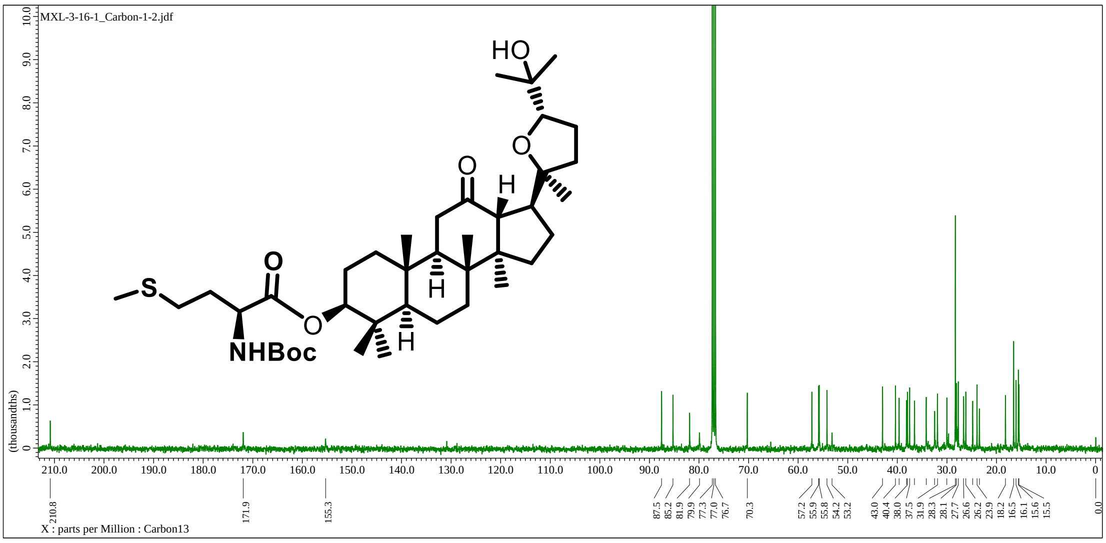

# $^{1}H$ and $^{13}C$ NMR Spectra of **9b**

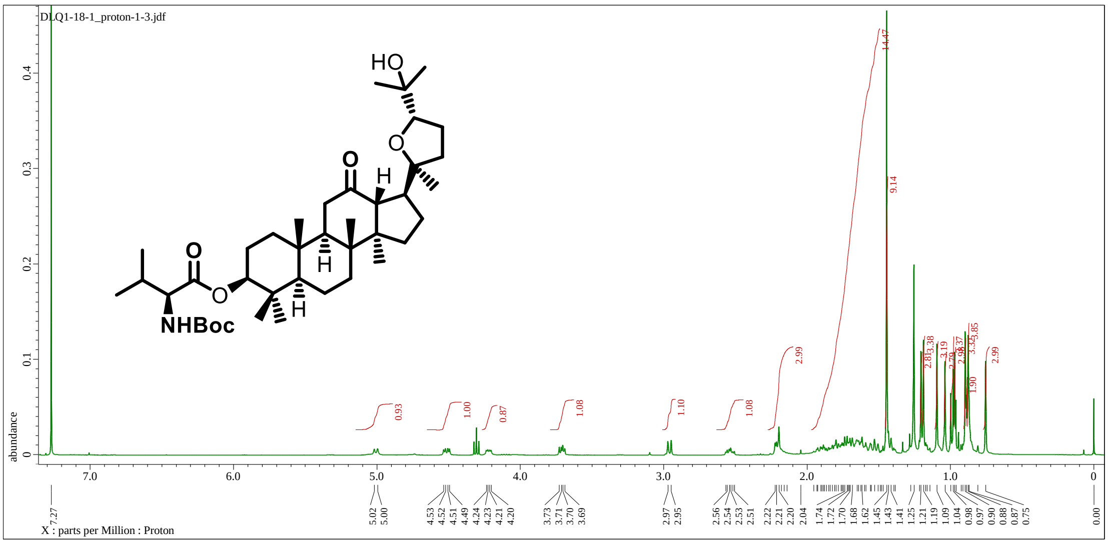


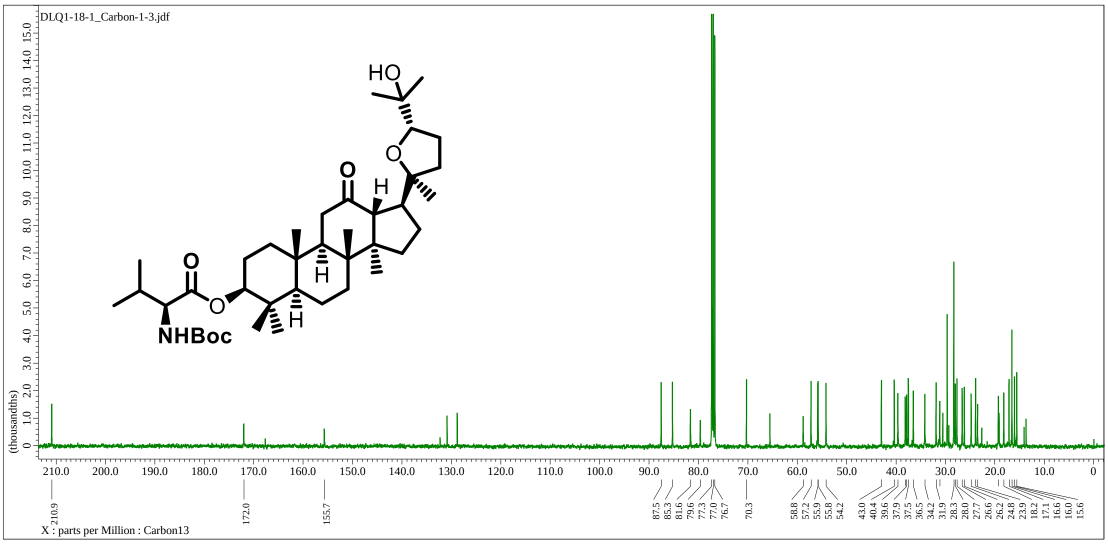

# $^{1}$H and $^{13}$C NMR Spectra of **9c**

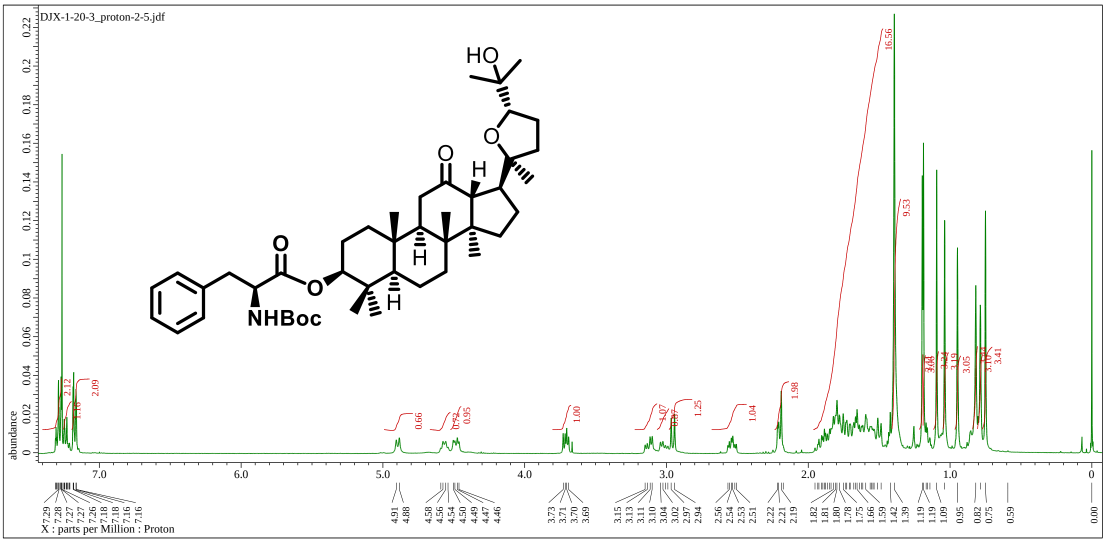


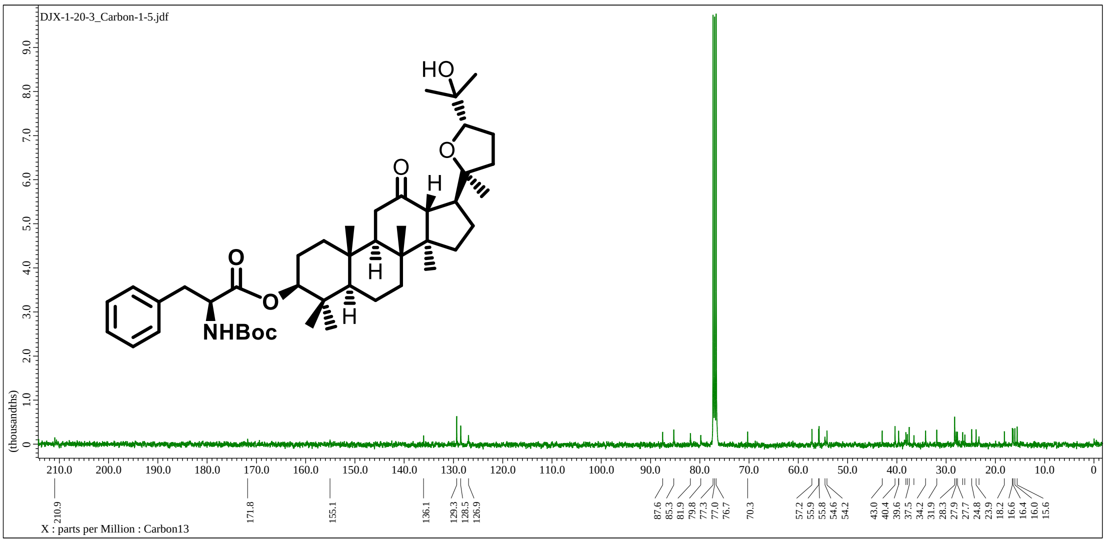

# $^{1}$H and $^{13}$C NMR Spectra of **9d**

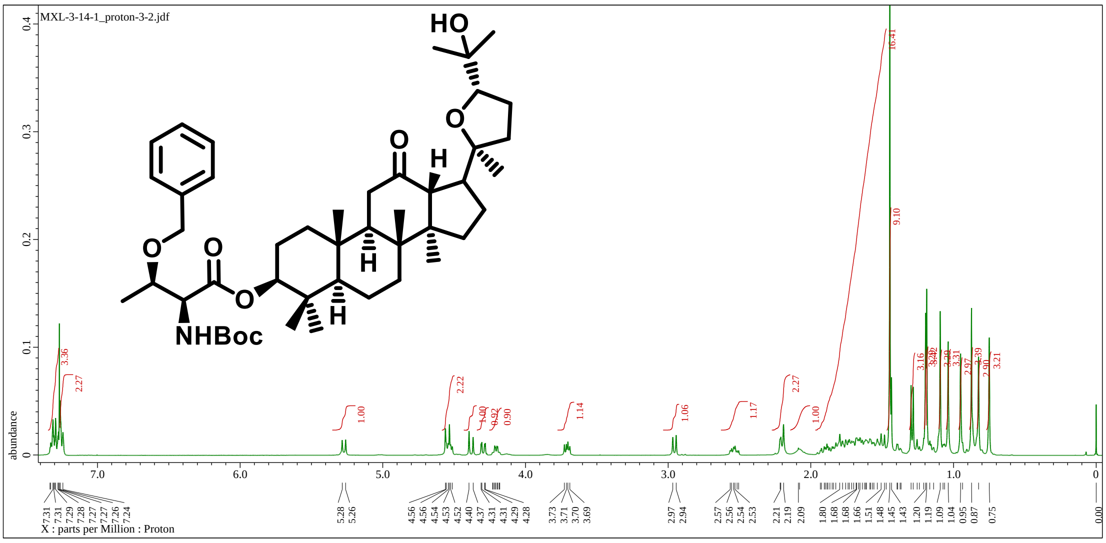


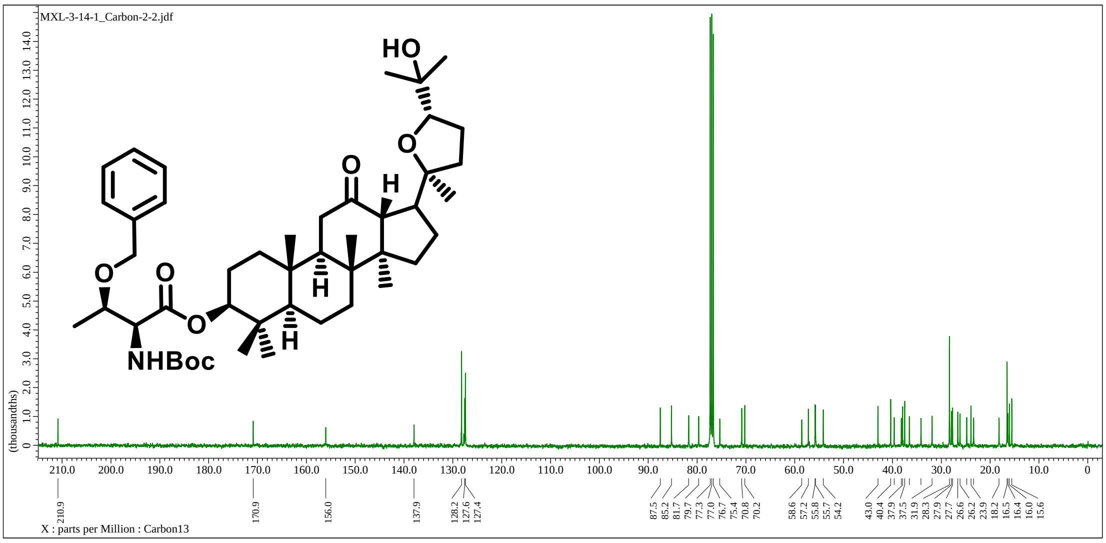

$^{1}H$ and $^{13}C$ NMR Spectra of **9e**

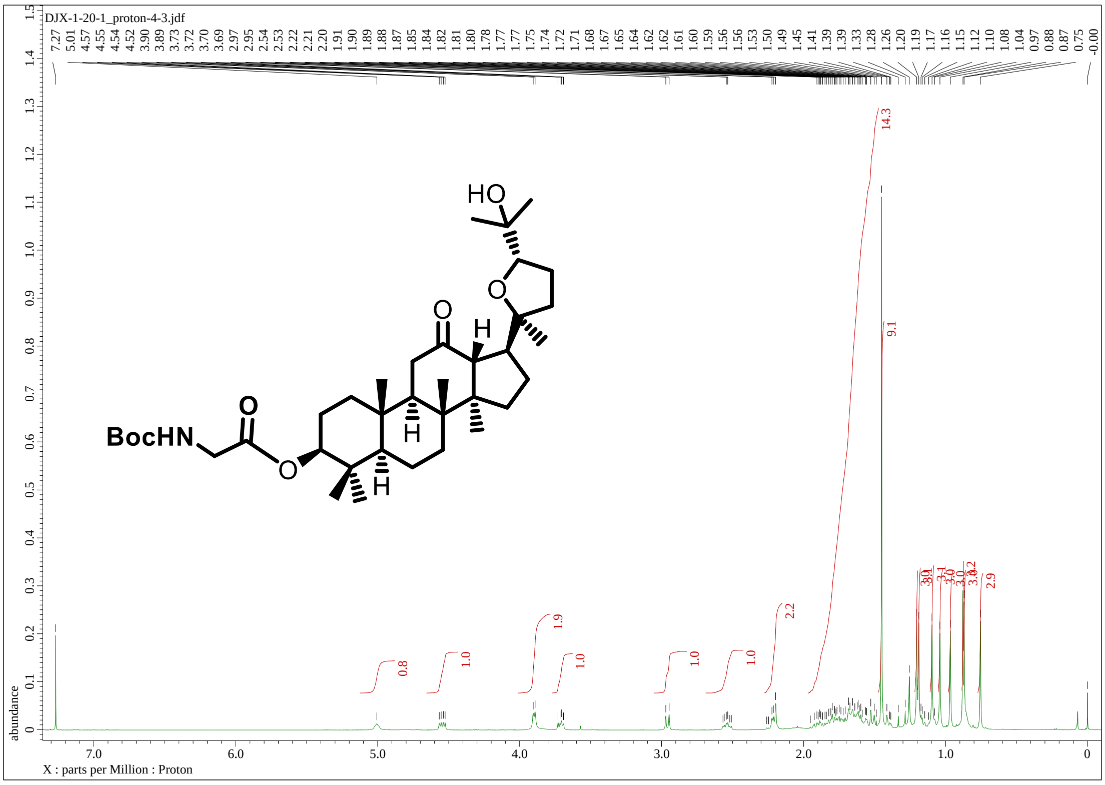

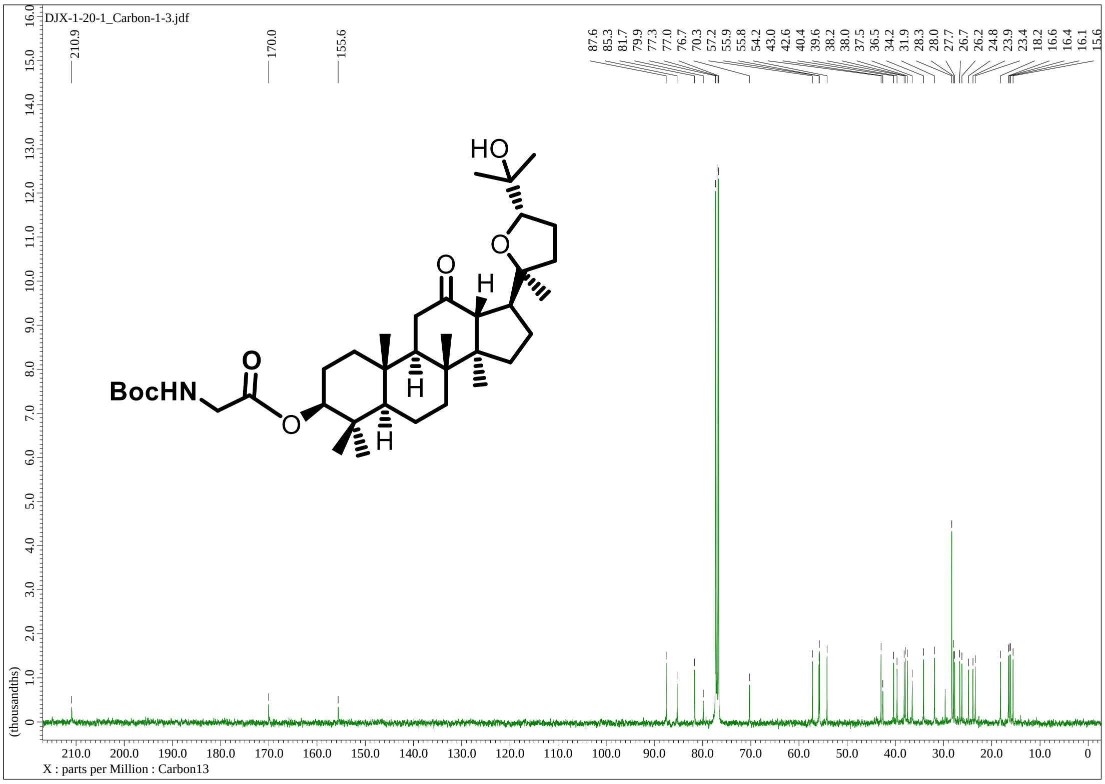

<sup></sup>

# $^{1}$H and $^{13}$C NMR Spectra of **9f**

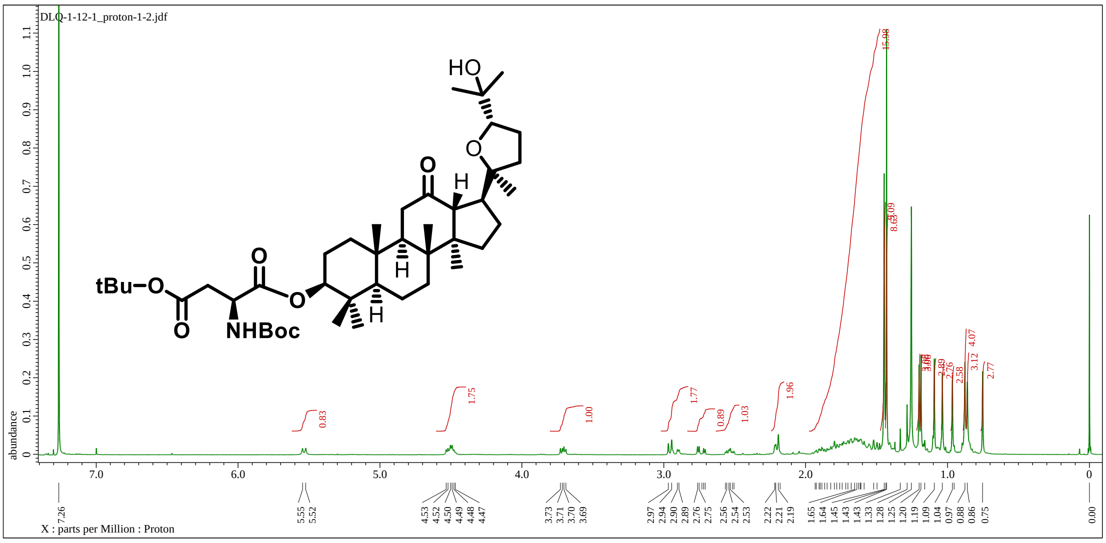

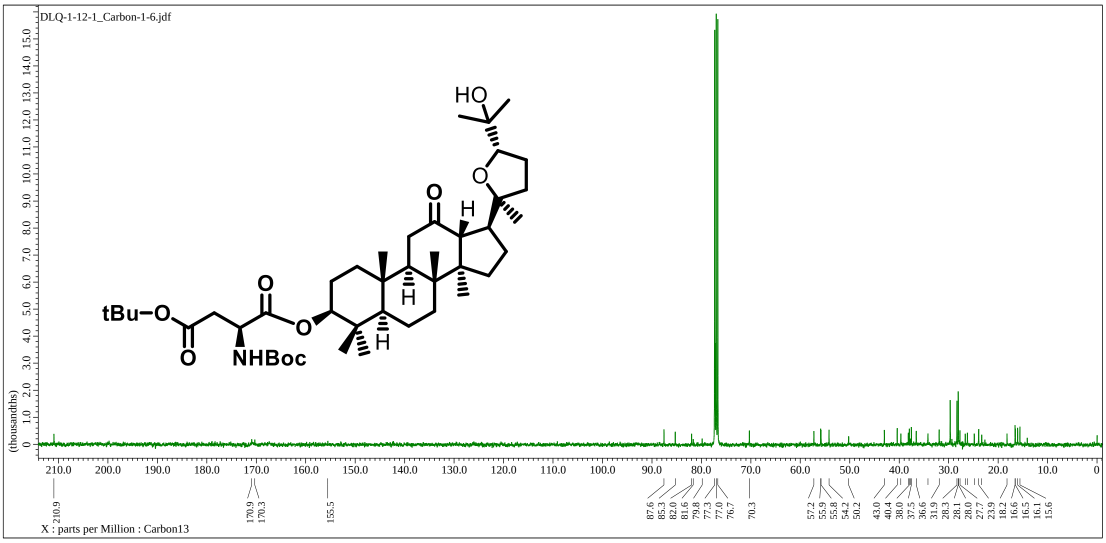

$^{1}H$ and $^{13}C$ NMR Spectra of **10a**

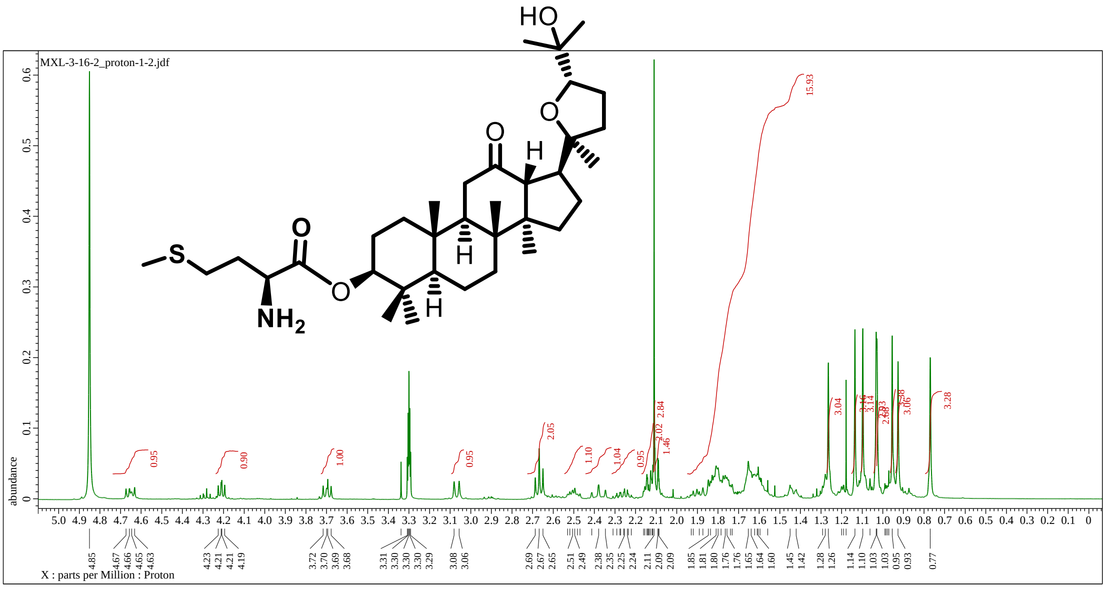

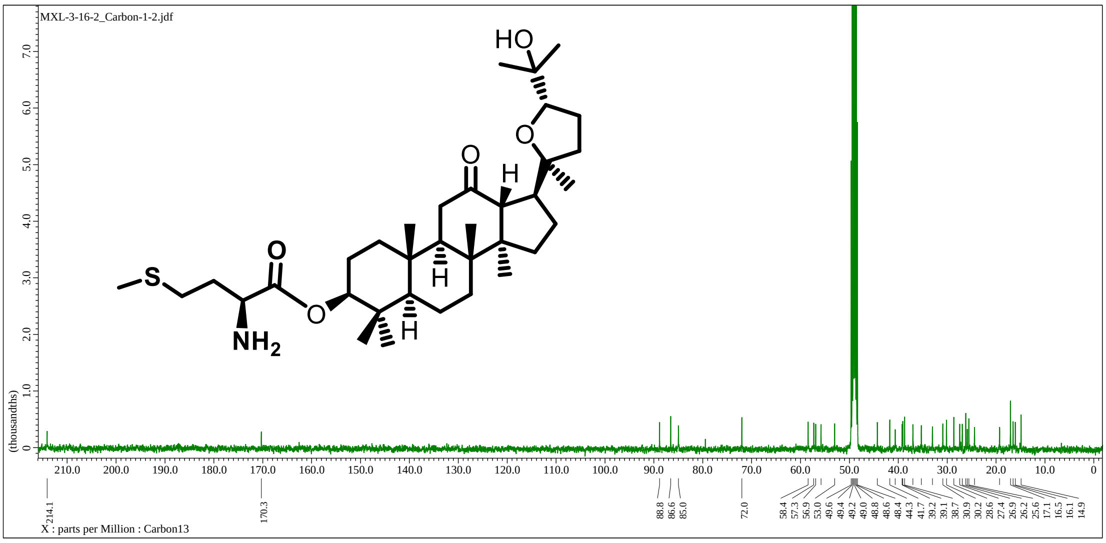

$^{1}$H and $^{13}$C NMR Spectra of **10b**

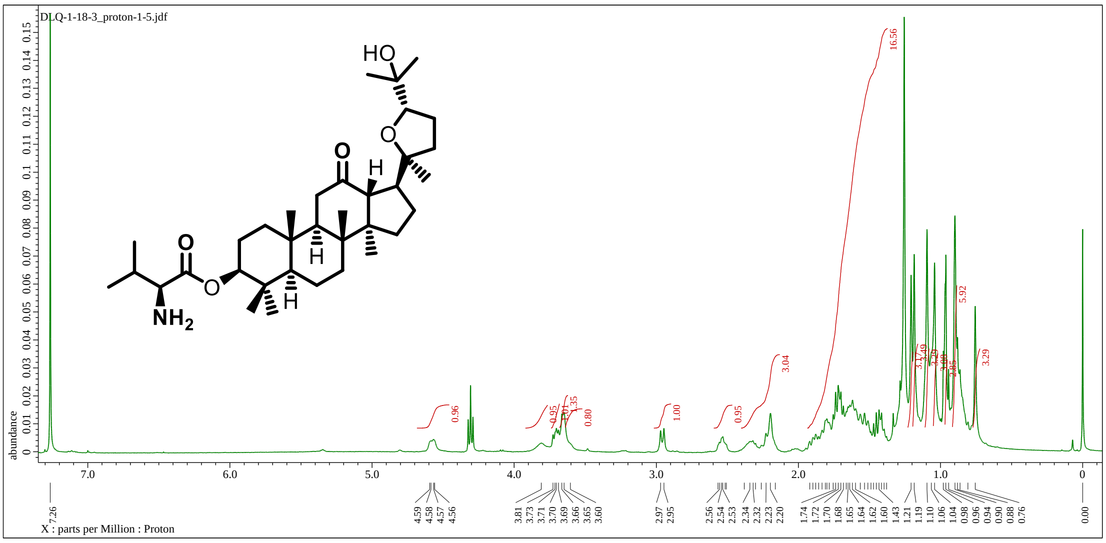


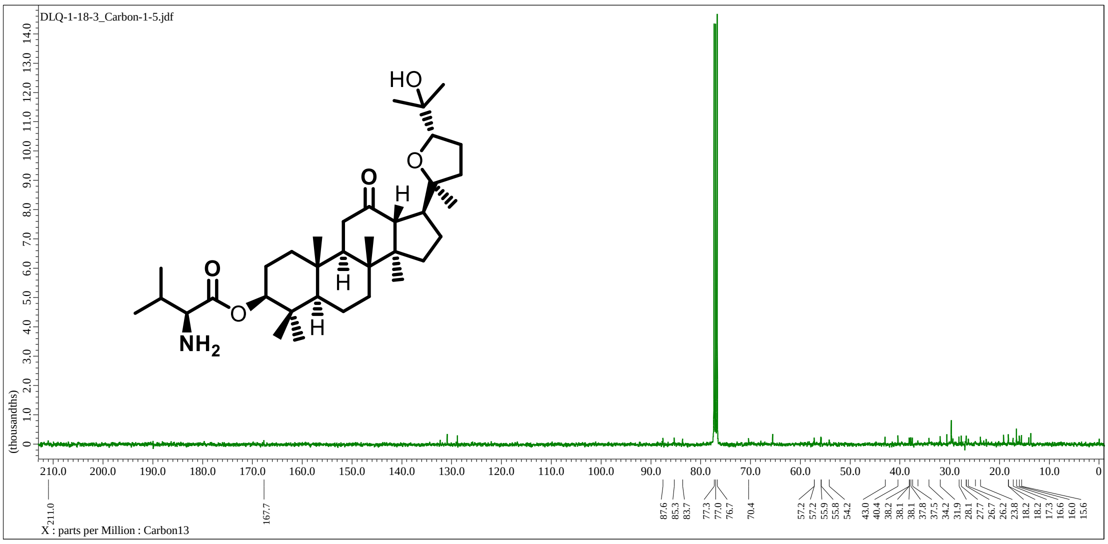

# $^{1}$H and $^{13}$C NMR Spectra of **10c**

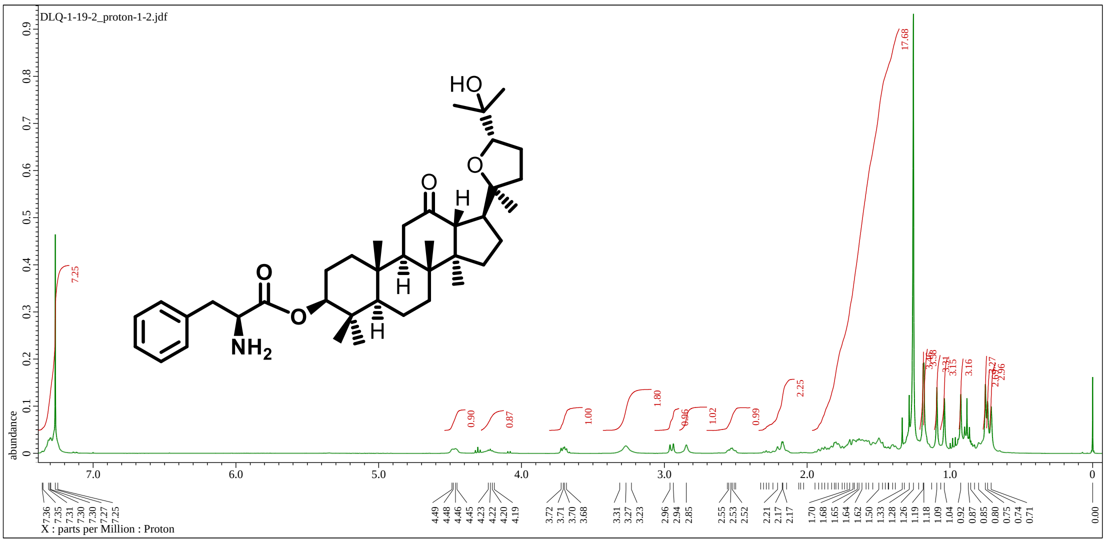


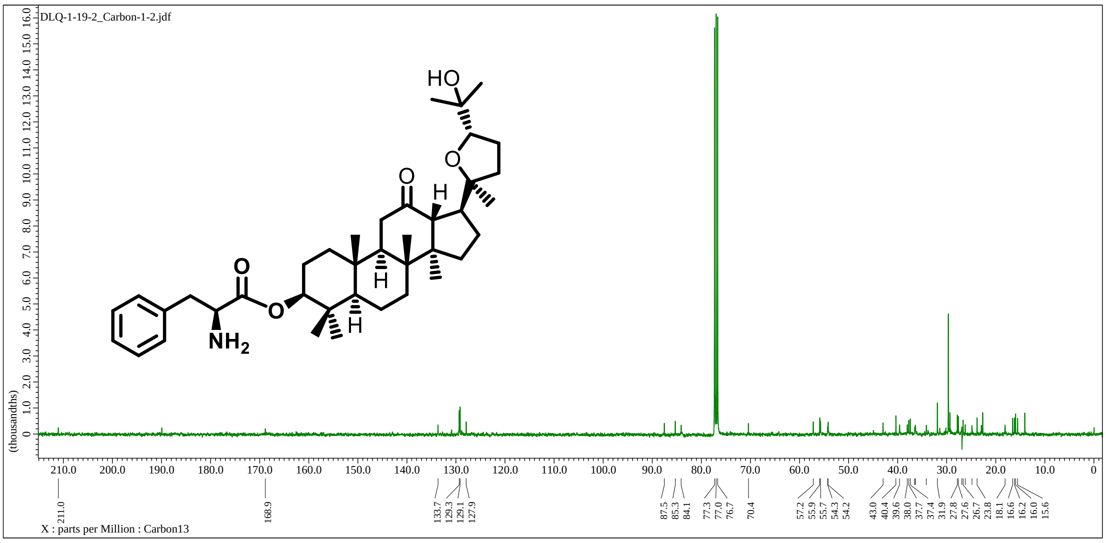

# $^{1}H$ and $^{13}C$ NMR Spectra of **10d**

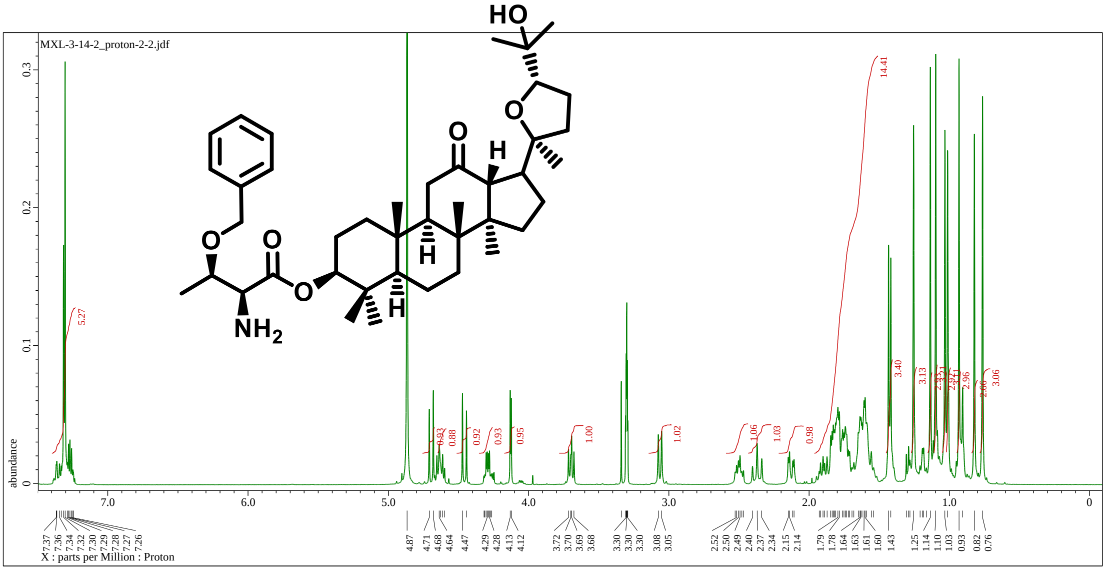


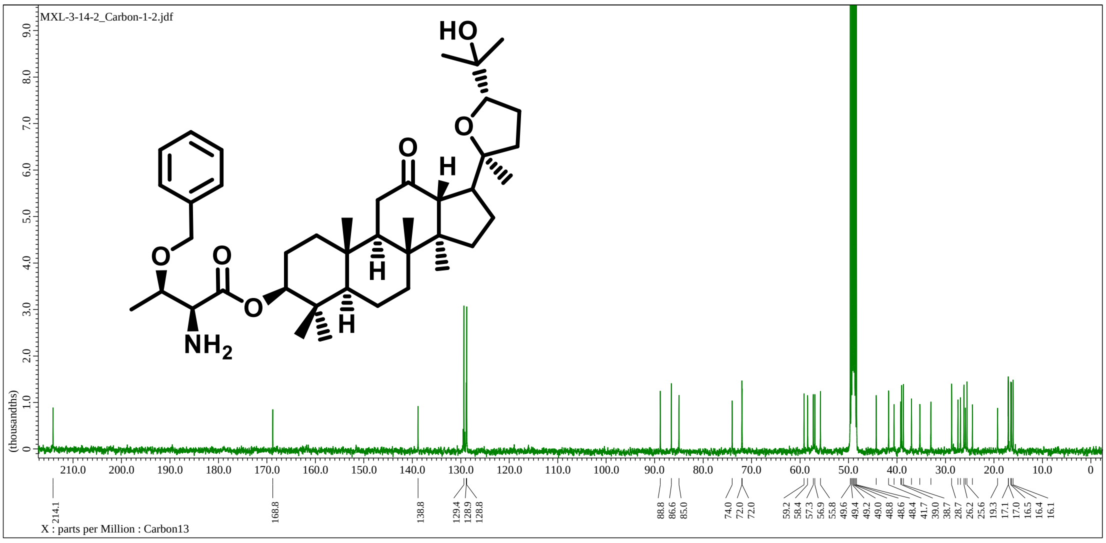

# $^{1}$H and $^{13}$C NMR Spectra of **10e**

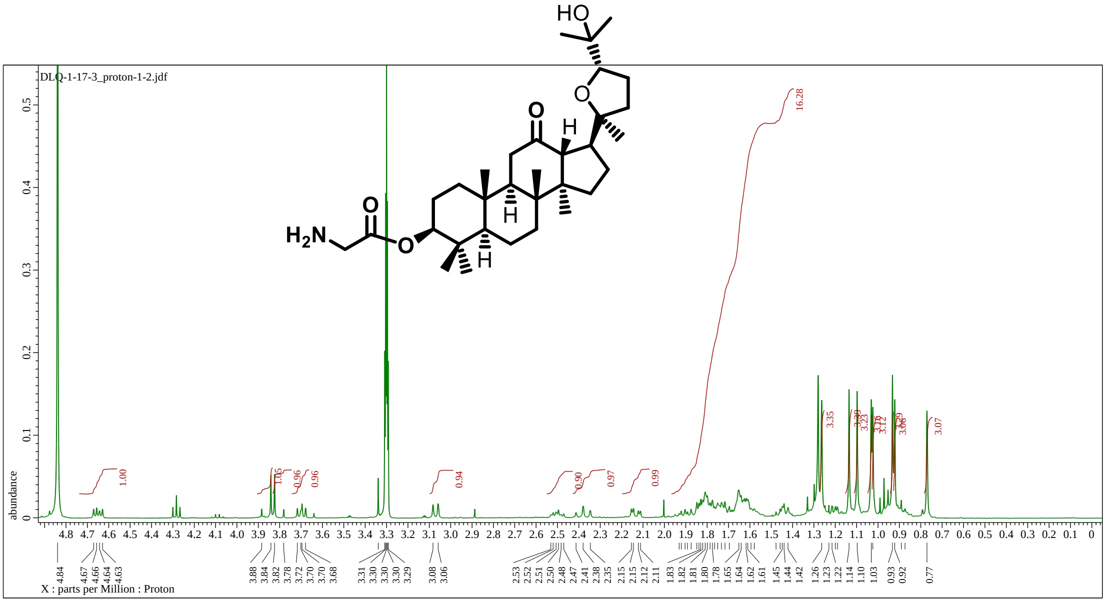

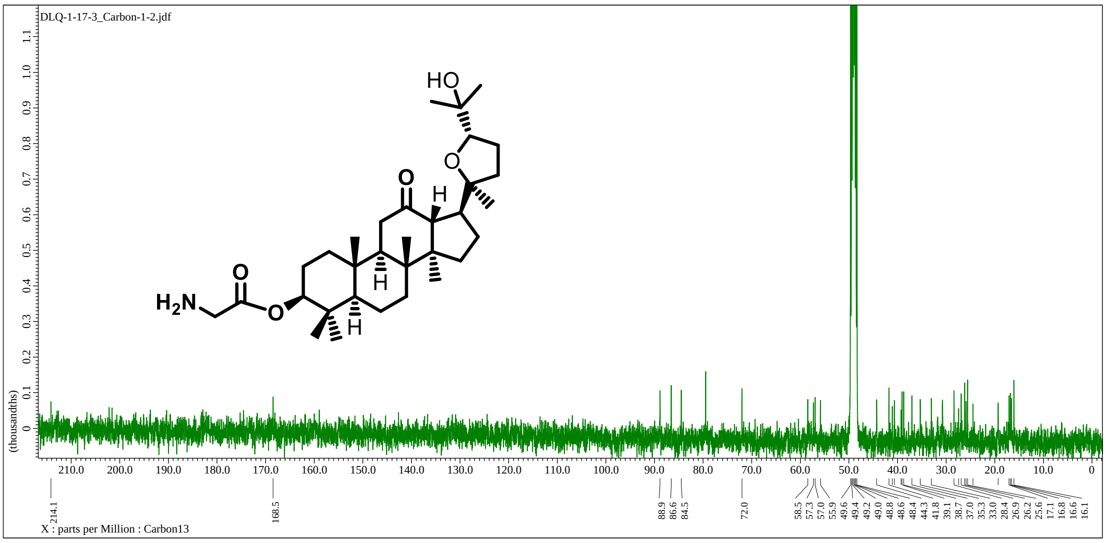

# $^{1}$H and $^{13}$C NMR Spectra of **10f**

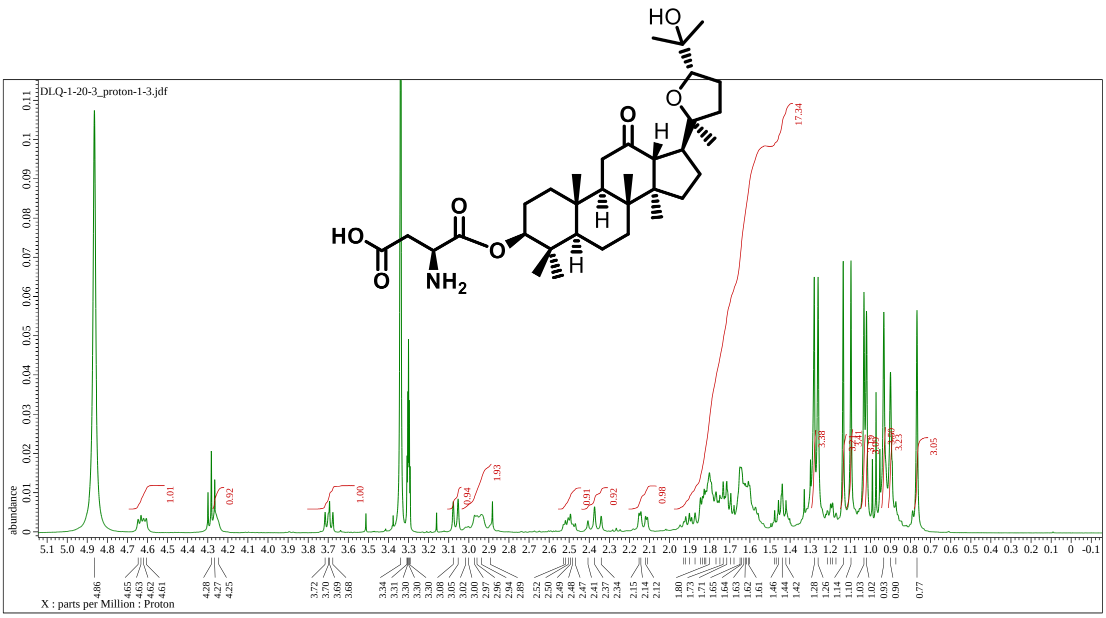


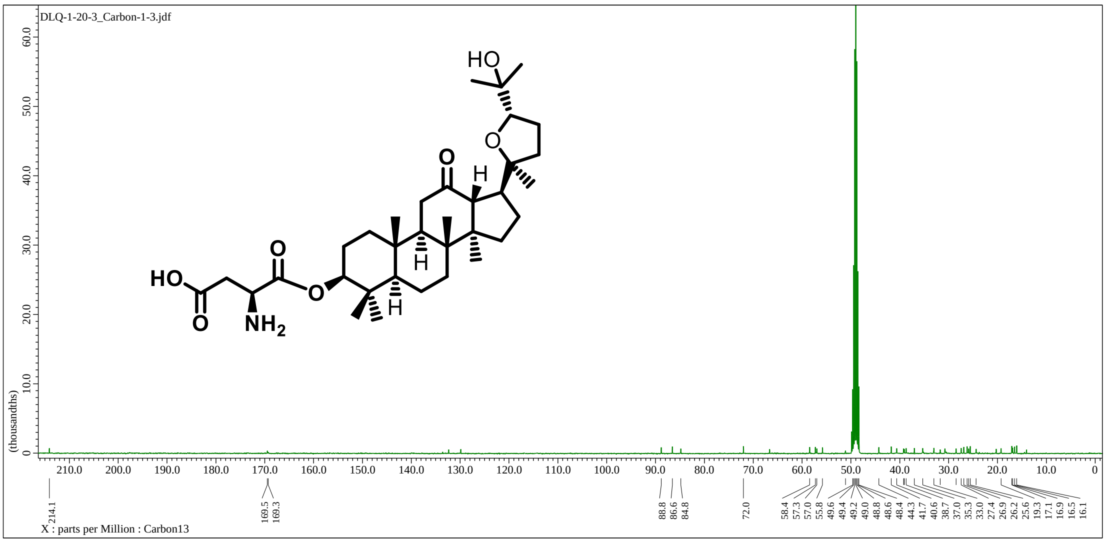

Supplement: Supplementary file 1 [file molecules-28-01307-s001.zip › molecules-2164081-supplementary.pdf]
